# Investigating the origin and authenticity of Victoria Cross medals using X-ray fluorescence spectrometry

**DOI:** 10.1038/s41598-020-76783-y

**Published:** 2020-11-19

**Authors:** Andrew Marriott, James G. D. Prendergast

**Affiliations:** 1 School of History, Classics and Archaeology, Armstrong Building, Newcastle University, Newcastle upon Tyne, NE7 1RU UK; 2 The Roslin Institute, University of Edinburgh, Easter Bush Campus, Midlothian, EH25 9RG UK

**Keywords:** Materials science, Scientific data

## Abstract

The Victoria Cross is the United Kingdom’s premier military award for bravery, presented for gallantry during active operations. Since its inception in 1856 just 1358 have been awarded, and, due to their rarity and historic interest, have become highly prized amongst private and public collections. Unresolved, however, is a debate about the source material of the medals. Some authorities adhere to a traditional belief that all medals have been cast from the bronze of guns captured from the Russians at Sebastopol. Furthermore, controversy is attached to the authenticity of some VCs. In this study we used X-ray fluorescence spectrometry data to compare the metal compositions of 100 Victoria Crosses, covering 7% of those ever issued. Using Gaussian mixture modelling we identify that Victoria Crosses fall into four distinct clusters, confirming that the primary split occurred between medals issued prior to and after 1914. Using these data we investigate the potential of X-ray fluorescence to inform the study of medals whose authenticity have been queried, showing some have unusually similar compositions to other VCs. This paper highlights how X-ray fluorescence data in conjunction with clustering approaches can be used to effectively and non-destructively investigate the authenticity and history of Victoria Crosses.

## Introduction

**Figure 1:**
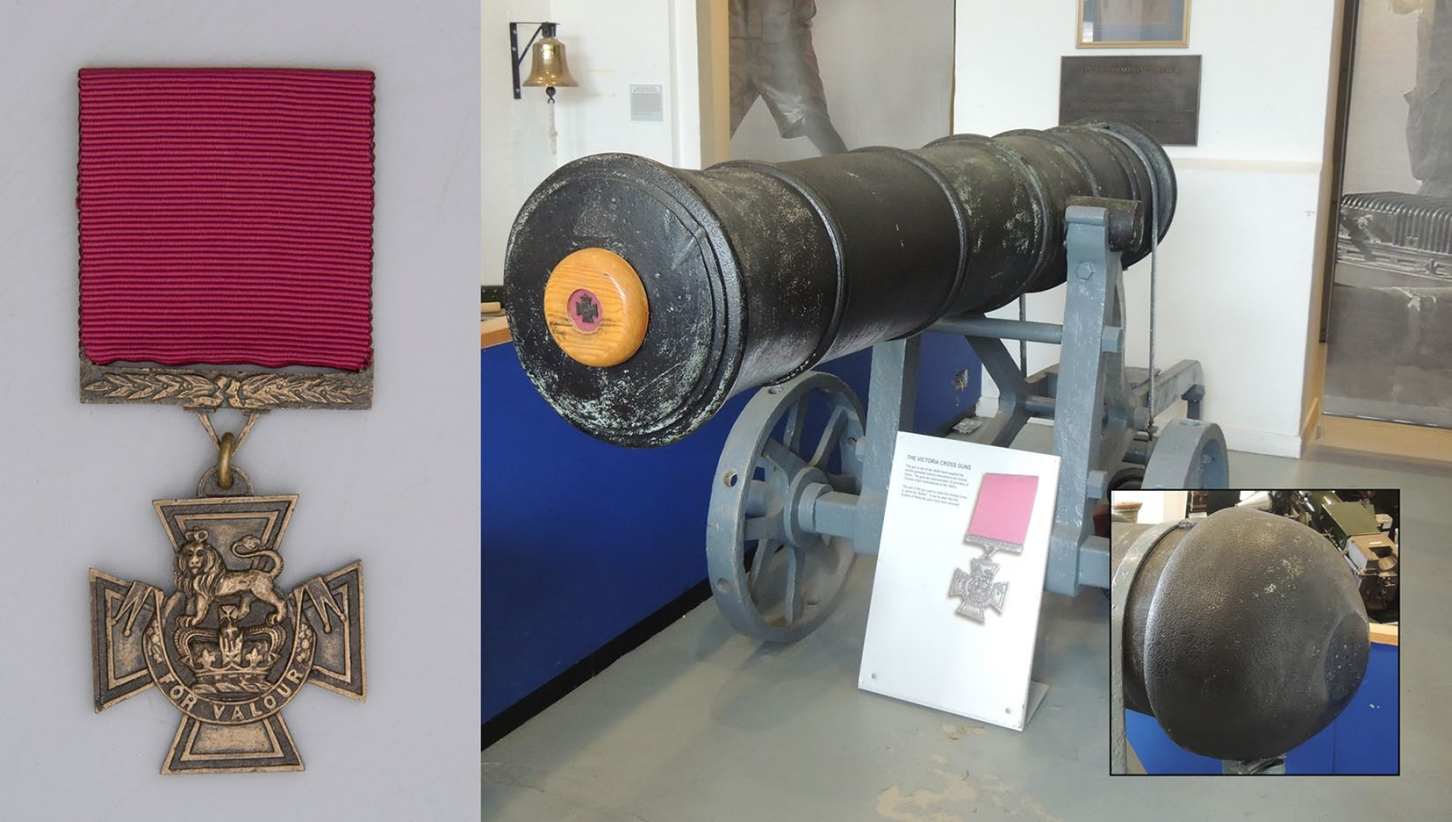
Left: A nineteenth Century VC (Innes). The date of the relevant action and the name of the recipient should be inscribed on the rear of the medal and the suspender bar. (Kind permission of the Royal Engineers Museum.) Right: One of the ‘VC Guns’ displayed at the Royal Artillery Firepower Museum with (inset) cut at rear of barrel showing cascabel to have been removed (author’s own image. Kind permission Firepower).

The Victoria Cross (VC) (Fig. 1) was instituted by Royal Warrant on 29 January 1856, initially recognizing acts of valour of British service personnel during the Crimean War, 1854–1856. Open to all ranks, it is awarded for acts of the most conspicuous bravery, some daring or pre-eminent act of valour or self-sacrifice, or extreme devotion to duty in the presence of the enemy^1^. To date, there have been 1358 awards and supply of the medals has only ever been entrusted to the London jewellers, Hancocks. As well as their intrinsic and commercial value, the medals attract academic interest regarding their source material and their provenance^2,3^.

The Royal Warrant stipulated that the medals were to be of bronze and a tradition became established that the bronze was sourced from guns captured from the Russians when the port of Sebastopol fell at the close of the Crimean War. There is, however, no corroboration for this belief beyond a comment in the *The Times* (27 June 1857), whose correspondent, reporting on the first presentation of medals by Queen Victoria, stated that they were “formed from the gun metal of ordnance captured at Sebastopol”. That notwithstanding, it is clear that Hancocks were being provided with bronze, or gunmetal, by the War Office from supplies at the Royal Arsenal, Woolwich, in order to make the medals^4^. Many authorities do hold to the assertion that all VC metal originates from captured bronze cannon, whose cascabels have been removed in order to provide the supply^5,6^. A cascabel is the rounded protrusion at the rear of muzzle-loaded cannon to which ropes or tackle could be attached. A pair of guns with removed cascabels were displayed by the Royal Artillery Firepower Museum (closed since 2016) as the ‘VC Guns’ (Fig. 1), but the origin of these guns and any association with Sebastopol has proved contentious^7^.

Post-1945 VCs have been cast from metal that, due to its shape, almost certainly once comprised part of a cascabel. That piece is held by the Ministry of Defence under secure conditions at a logistics depot in Donnington, Shropshire, cuts being provided to Hancocks as required^8^. The ‘VC Guns’ are thought unlikely to have had their cascabels removed until 1914^7^. The Donnington piece is unlikely to have come from either of these ‘VC Guns’. Rather, if used, their cascabels would have been exhausted in supplying the 628 awards of the Great War and many of the further awards of the Second World War. Thus there are at least three candidate sources of VC metal post-1914, and potentially a range of others since 1856, with the possibility of the Sebastopol association being entirely apocryphal^9^.

By the beginning of the twentieth century, VCs had attracted the interest of collectors and were becoming marketable commodities. In 1900 a medal attributed to Private O’Hea for an action in 1866 was sold at Debenhams, London, for £57. That VC was subsequently declared to be a counterfeit, the original being in Australia. The Australian medal was examined and authenticated by Hancocks in 1907 and the case caused the War Office to require that all subsequent medals be marked on the reverse in a manner known only to the jewellers^10^. Hence the provenance of all later medals could be verified through Hancocks’ records.

Verification of the earlier VCs remains problematic, being dependent upon visual examination and subjective determination by medal experts. By the 1980s, however, the UK Royal Armouries, using X-ray fluorescence, developed a programme of investigation of VCs which was able to determine the detailed profile of the elements present in each medal; essentially the medal’s composition fingerprint. While the results have never been published, the Royal Armouries had confidence in their ability to determine authentic medals from cast copies. Most notably, they resolved that the VC won by Lt John Chard at Rorke’s Drift during the Zulu War of 1879, and purchased as a copy by the actor Stanley Baker in 1972, was, in fact, the original medal presented to Chard (Daily Telegraph 13 June 1996). More recent examination of a corpus of VCs was conducted by one of the authors^11^. This included X-ray fluorescence of two 19^th^-century medals whose provenance was in question and they are discussed below. Notably, the metallic profiles of those medals presented very close correlates with their contemporaries from the Crimean War and the Indian Rebellion of 1857.

### X-ray fluorescence

X-ray fluorescence (XRF) spectrometers can be deployed as entirely non-destructive investigative instruments and are particularly appropriate for use on rare or special artefacts curated in environments such as museums. Modern equipment is portable and it can provide almost immediate and accurate data regarding the composition of alloys such as bronze. The process works by emitting a photon beam upon the object. The interaction of the primary X-ray beam with the sample’s atoms excites the atoms’ electrons, causing some electrons to be knocked out of their orbits: this leaves a vacancy and causes a temporary state of instability in the atom. This instability is corrected as electrons from higher energy orbits replace the displaced electrons. This event produces an energy that is specific to the atom of each element. Thus the emission signatures from various elements allow the identification of which may be present; quantitative data can be established from the number of energies produced by each element.

XRF has previously been used to examine the composition of VCs beyond just the study undertaken by the Royal Armouries, with subsequent analyses of medals in Australia and New Zealand confirming their composition of predominantly copper and zinc^2,3^, though the precise ratio of these metals was observed to differ between medals. In this analysis we have compiled composition data for the largest set of VCs to date to investigate potential changes in composition over time and the characteristics of medals of queried provenance.

### The data sets

One set of XRF data are those collected by Marriott between June 2016 and January 2018^9^. They included fifty Victoria Crosses, spanning examples from the Crimean War (1853–1856), the Indian Rebellion of 1857, the Boer War (1899–1902), WW1 (1914–1918), WW2 (1939–1945), the Falklands War (1982) and recent operations in Afghanistan starting in 2002. Also examined were the cannon displayed as the ‘VC Guns’ at the Royal Artillery Museum, Woolwich, the cascabel piece at Donnington and an early proof medal.

The second set of data has been taken from notes and summaries provided by the Royal Armouries of their XRF investigations of the 1980s and 1990s. They include seventy-one VC medals ranging from the Crimean War to the Falklands War, of which three were identified as copies or duplicates. Two likely authentic medals were excluded from the current study (RM 1854 and HVC 1854) as their zinc compositions had not been recorded. XRF data was also gained from the ‘VC Guns’ and the Donnington cascabel piece as part of the RA’s analyses. Additionally, the Royal Armouries had collected data from Hancocks, including five unissued VCs, various samples of VC metal and a piece described as a casting tree.

Eight medals were common to both data sets. While up to four medals may have been duplicates, the sample is believed to be based upon 110 VCs, of which the provenance of two remain to be confirmed.

## Results

### Comparing the datasets

As eight medals had been analysed by both the Royal Armouries in the 1980s and Marriott in 2018 this allowed us to first analyse how consistent the measurement of metal composition was across the two different studies. For most items in the Marriott study two readings had been taken so that the consistency of measurement could also be analysed within this specific study. Eight medals had a Pearson’s correlation coefficient of less than 0.95 between Marriott repeat measurements and these were first removed from all further analyses.

**Figure 2:**
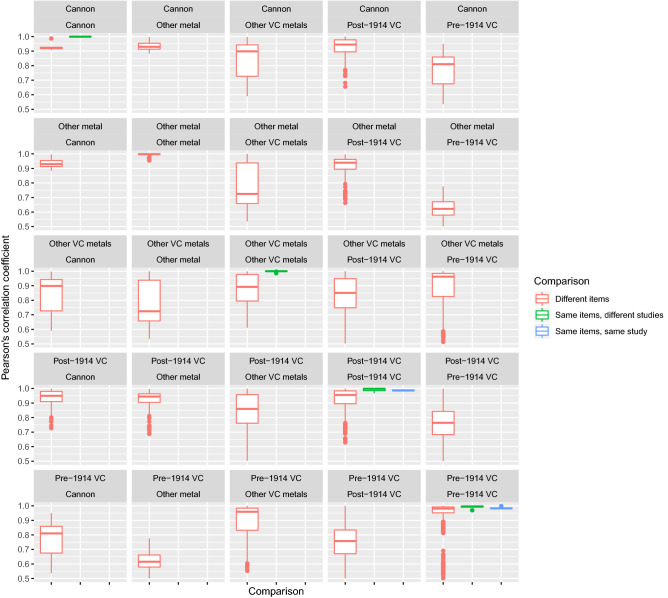
Comparisons of the metal compositions of different groups of items within and between studies. Each plot represents all pairwise Pearson’s correlation coefficients between the log transformed metal compositions of groups of items. The median (central horizontal line), first and third quartiles (box ends), 1.5 × interquartile ranges (ends of whiskers) and remaining outliers (dots) are indicated. The comparisons in each panel is split into three as defined in the legend and the groups of items being compared are indicated by the labels above each plot. The y axis was truncated at 0.5.

Items were grouped into six categories (Supplementary Table S1). Medals awarded for actions before and after 1914, other VC metals (for example the VC casting tree from Hancocks, unissued VCs and official replica medals), cannon and finally other metals. The latter category included the blocks of source metal stored at Hancocks and Donnington. As shown in Fig. 2 there was a high consistency in the quantification of metal composition between the repeated measurements across the two different studies. On average the Pearson’s correlation coefficient between the natural log transformed metal compositions of the medals measured in both studies was 0.99, with a minimum correlation of 0.98. In contrast the corresponding correlation between randomly chosen medals issued prior to 1914 was 0.96, and 0.93 for pairs of medals issued after 1914. So although medals generally show similar compositions, the repeat measures for the same medal across the two studies thirty years apart are unusually strongly correlated, consistent with these analyses having produced accurate and comparable measurements of metal composition.

### Clustering of medals by age

**Figure 3:**
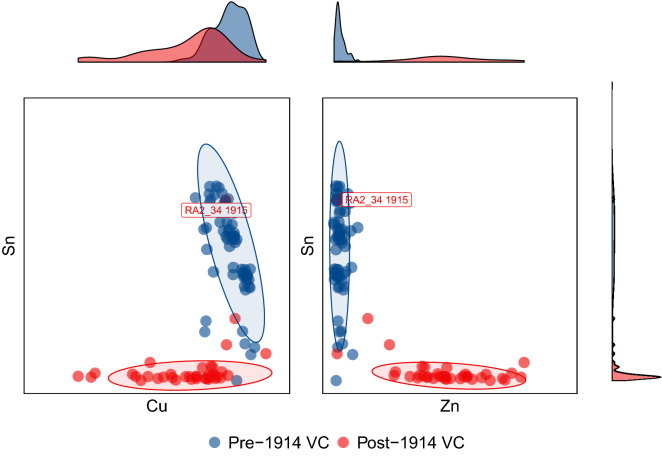
The separation of early and late VCs by composition. The tin, copper and zinc compositions of Victoria Crosses and associated metals. 95% normal confidence ellipses are drawn around the pre and post 1914 VCs (blue and red respectively) with marginal density plots indicating the distribution of metal compositions of each group shown along each axis. The composition of the RA2_34 VC is indicated.

Previous analysis of the Royal Armouries dataset suggested that VCs issued before and after 1914 show distinct metal compositions^7^. Consistent with this, the correlations between the metal compositions of medals from before and after this date is generally a lot lower than observed within eras (Fig. 2). To investigate this further, and to examine whether this was also the case across the newly collected data, we contrasted the metal compositions of the VCs issued before and after this date. As shown in Fig. 3 pre-1914 VCs are associated with comparatively high copper and tin levels whereas those issued after 1914 contain little tin but higher levels of zinc. This is consistent with the hypothesis that the source metal for Victoria Crosses changed around the beginning of the First World War. The medals from these different eras largely separate based on their composition of these metals. A clear exception is though observed, with one of the VCs from the Royal Armouries analysis dated as from 1915 clustering with the pre-1914 medals. The Royal Armouries composition data for this medal was not, however, associated with the name of its recipient making further investigation difficult.

As shown in Supplementary Fig. S1A the differences in compositions between VCs of different eras is not just restricted to these primary metals. To refine this analysis and automatically partition the medals and associated metals into groups we undertook Gaussian finite mixture modelling of the data. This technique automatically partitions the data into groups of items that show similar compositions across the six elements examined (iron, copper, zinc, lead, tin, and arsenic). Importantly it is an unsupervised technique, meaning that the clustering is done blind to the type and age of items. It is therefore possible to subsequently compare the clusters defined by the model to the known annotations of the items, to examine the extent to which items of the same (or unknown) type cluster.

**Figure 4:**
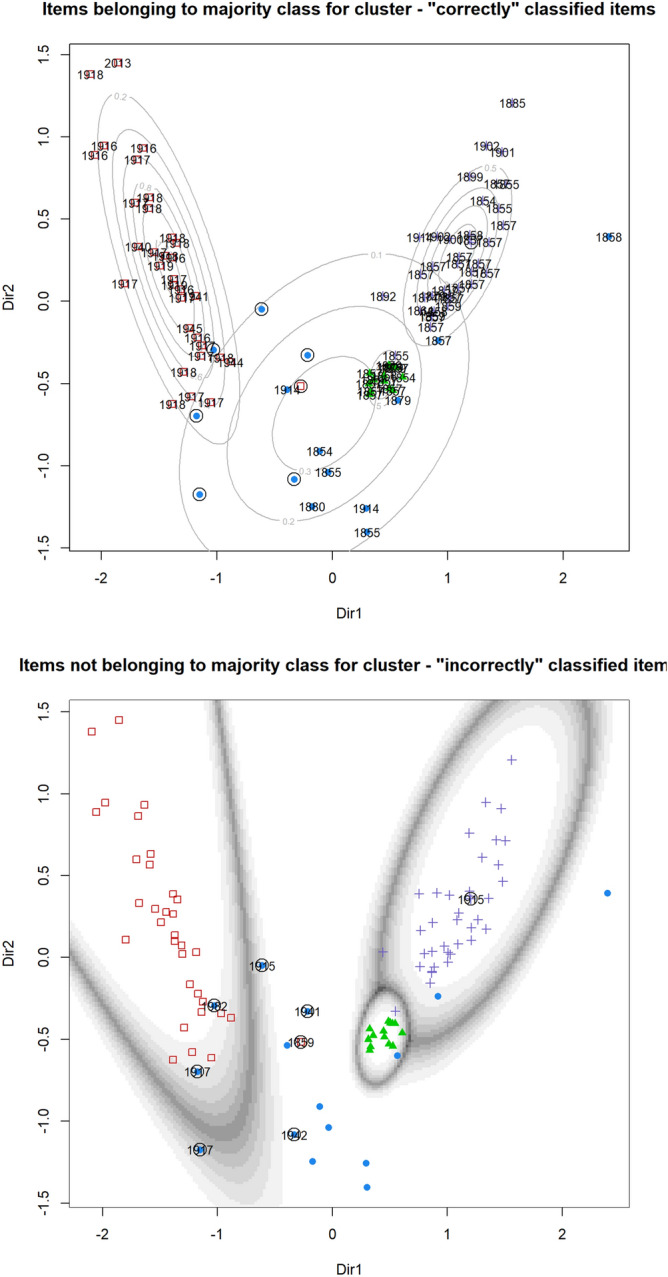
The four clusters of VCs defined by gaussian finite mixture modelling. Dimension reduction projection of the finite mixture modelling results. Point shapes and colours indicate the different clusters (blue: cluster 1, red: cluster 2, green:cluster 3 and purple: cluster 4). In the top plot items that match the majority type of that cluster are labelled with their corresponding date, with those not matching labelled in the plot below. In both plots the mismatching items are circled. In the top plot contour plots of the estimated mixture densities are shown. In the bottom plot the uncertainty boundaries of the clustering are indicated.

As shown in Table 1, Supplementary Fig. S1B and Fig. 4 this approach defined four distinct clusters (likelihood ratio test bootstrap p value of: 3 versus 4 clusters: 0.001; 4 versus 5 clusters 0.998). Two of pre-1914 VCs one of post-1914 VCs and a final mixed cluster. Among the 85 medals in clusters 2, 3 and 4, only two clustered with medals from different eras (i.e. pre or post-1914). The aforementioned RA2_34 VC of unknown attribution, and the medal awarded to Captain Herbert Clogstoun for his actions during the Indian Mutiny/First War of Independence in 1859 which has a composition most similar to the set of post-1914 era medals. Excluding these two medals the remaining medals within each of these clusters all originated from the same eras. All but one of the medals in the smaller of the pre-1914 VC clusters (cluster 3) was examined in the Royal Armouries analysis, in contrast to 53% of the larger cluster (cluster 4). It cannot be excluded therefore that the slight separation of these two clusters is the result of some form of batch effect despite the high correlations observed between the repeat measurements across the two studies. The final cluster of fifteen medals contained a mixture of those from both eras.

**Table 1:** Statistics of the four clusters defined by finite mixture modelling.

Cluster	1	2	3	4
No. of pre-1914 VC	9	1	14	37
No. of post-1914 VC	6	32	0	1
Median date	1914	1917	1857	1857
Mean date	1899	1921	1862	1869
Date St. Dev	39.6	21.3	9.48	19.4
Prop. measured by RA	0.33	0.64	0.93	0.53

**Figure 5:**
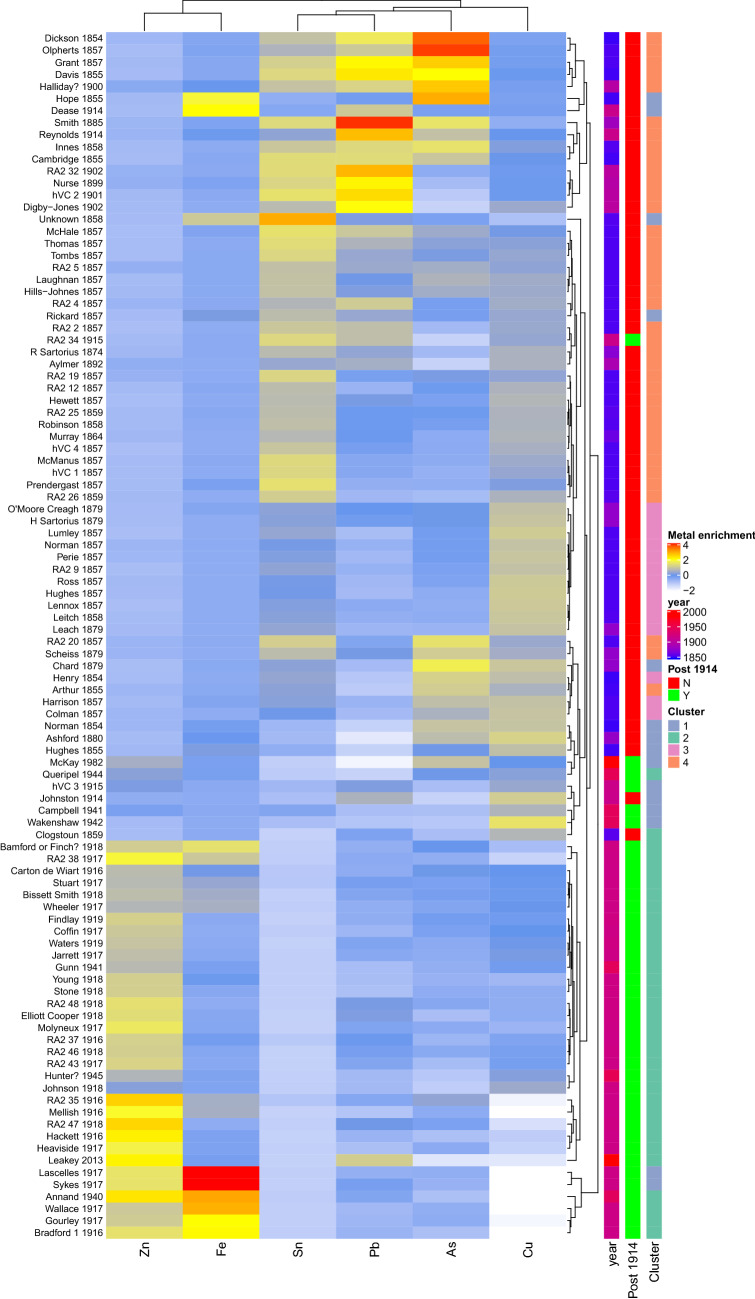
Heatmap indicating the clustering and relative compositions of the VCs. Medals clustered according to the Euclidian distance between their compositions. Compositions were scaled so that the mean in each column is 0 and the standard deviation is 1. A low metal enrichment value indicates the corresponding medal has an unusually low composition of that metal relative to the other items analysed. The year, era and finite mixture modelling cluster of each VC is indicated on the right of the plot with the relationship between items within a cluster indicated by the dendrograms.

To pick up finer relationships between the samples we also performed hierarchical clustering of the medals (Fig. 5). This largely recapitulates this separation of samples, however, composition differences between even medals of the same era and cluster can be distinguished in this analysis. For example, the medals of Lascelles, Sykes, Wallace, Gourley and Bradford dating from 1916 to 1917 form a sub-cluster with the medal of Annand dating from 1940. These medals display an unusually low copper content and higher iron content as compared to other medals dating to after 1914.

One potential explanation for these observed sub-clusters is that they reflect medals of different batches generated at different times with potentially differences in source material and/or casting approaches. Broadly consistent with this the medal awarded to Lieutenant Annand mentioned above was the first awarded during the Second World War, and therefore potentially remaining from a previous cast of medals.

In this Figure, RA2_34 and the medal of Captain Herbert Clogstoun are again clear outliers. Although a third medal, awarded to Captain William Johnston, clusters with later era medals, this medal being dated to 1914 sits at the dividing line between the two eras and may therefore simply represent the switch between sources during this year.

### Exploring the source metal of the medals

As discussed, there remains debate as to the source metals of the VCs and the clear split in composition of pre and post-1914 medals suggests the source has likely changed at least once. To explore this further we compared the composition of the medals to that of five blocks of metal supplied by the Ministry of Defence to Hancocks and the block of metal held at the MoD base in Donnington, as well as the “VC guns” held at Woolwich. To enable the comparison of whether the composition of the “VC guns” was unusually similar to that of the medals as well as the Hancocks and Donnington blocks, we also included composition data for cannon found aboard seventeenth and eighteenth century Dutch shipwrecks found off of the coast of Australia^12^. As these bronze cannon cannot have been the source material of the medals they are an effective control to compare how similar the “VC guns” are to the medals and blocks.

**Figure 6:**
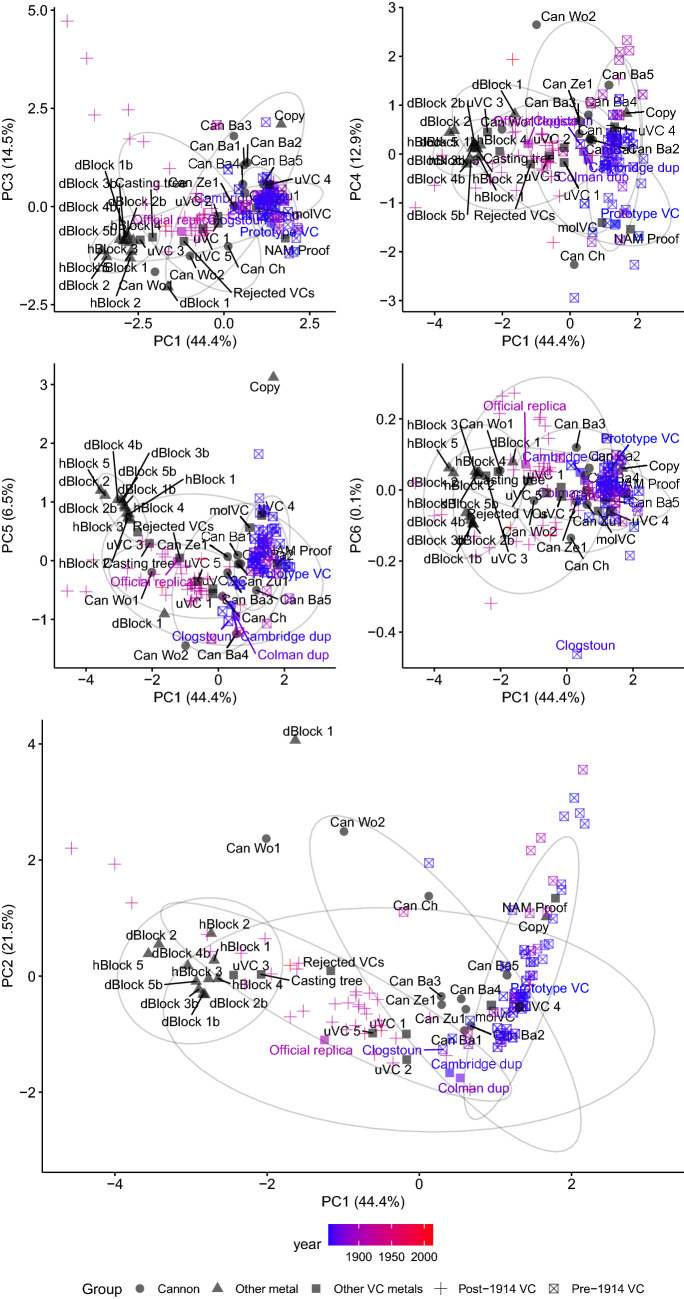
Principal component analysis of the medals and associated metals. Principal component 1 (PC1) versus each of the other five principal components are shown in separate plots. The VCs are coloured by their age. Non-VC items and the Clogstoun VC are labelled. Can Ba, Can Ze and Can Zu correspond to the cannon from the three Dutch shipwrecks. dBlock correspond to Donnington blocks, hBlock refers to Hancock’s blocks and Can Wo and Can Ch the “VC guns” as measured by the Royal Armouries and Marriott respectively. 95% normal confidence ellipses are drawn for each item group.

Principal component analysis (PCA) of these data (Fig. 6) illustrates that the Hancocks and Donnington blocks are of similar composition, and that both cluster near to the post-1914 medals. This supports the idea that the Donnington block is the source material of the more recent medals. Notably, however, the Woolwich cannon do not show particularly similar composition to these blocks nor the medals of either eras. Perhaps surprisingly, the cannon found on the Dutch shipwrecks show compositions more similar to that of the medals, and in particular the earlier medals from the nineteenth century. Although the composition of cannon can differ along their length this raises the question as to whether the Woolwich cannon have ever been the source of the VC metal.

### Using XRF to investigate the authenticity of medals

A common issue with Victoria Crosses are questions regarding the authenticity of particular medals. This can arise for a number of reasons and one example of this was the discovery of a medal inscribed with the date “5 NOV 1854” in the river Thames in London in 2015^13^. Understanding the composition of a medal of unknown provenance could inform its potential authenticity. Although approaches such as the use of machine learning would likely be a valid approach for separating genuine VCs from other items, there is currently insufficient data on items that are not genuine medals to effectively train such approaches. An alternative approach is to simply identify the distance of the composition of items from authentic medals to provide an indication of how typical an item’s composition is for a medal of a certain date.

For those 8 years where there were four or more medals in the dataset (1854, 1855, 1857, 1858, 1879, 1916, 1917 and 1918) we used the PCA results to calculate the weighted squared Euclidian distance between each item in the dataset and the median composition of all the medals issued in that year. So for example, for a given medal the squared difference of its value on PC1 to that of the median PC1 value for all medals issued in 1854 was calculated (excluding the medal from the latter if it was dated to that year). These distances were then multiplied by the proportion of variance explained by the corresponding principal component in order to upweight the more informative PCs. See methods for further details. These distances across all principal components were finally summed providing a metric of how far the item is from a typical medal dated to a given year. The smaller this distance metric the more typical the item’s composition is for medals issued in that year. Calculating the metric in this way, i.e. with respect to the different years, has the advantage of mitigating the potential impact of batches. For example, just calculating the distance to all medals of a given era may lead to a loss of resolution given the previous evidence of potential sub-clusters within the data (Fig. 5).

**Figure 7:**
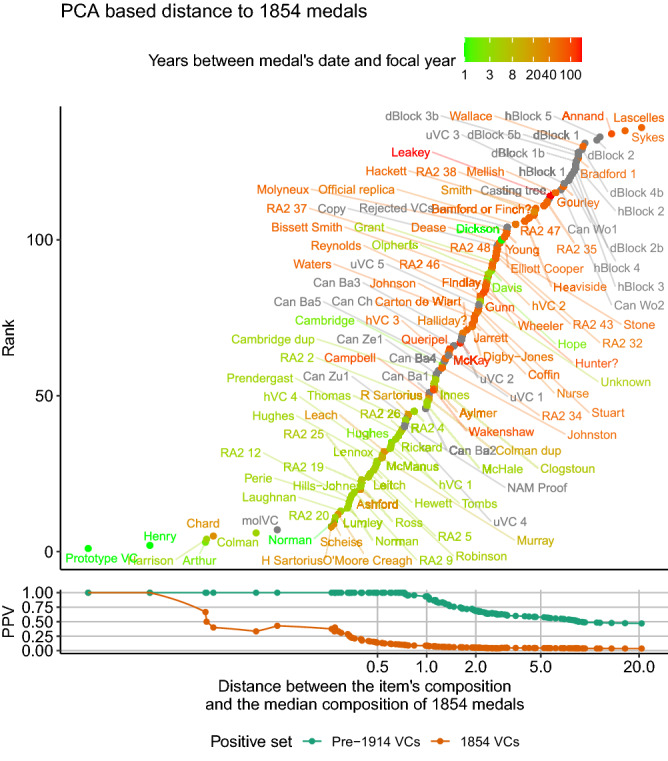
The similarity of items to the group of medals issued in 1854. The weighted squared Euclidian distance between the metal composition of each item and the median composition of all medals issued in 1854 (the medal in question being excluded from the calculation of the median composition if issued in that year). Items in the top panel are shown ranked by their distance and the colour of their points indicates the years between the year in question and the date of the medal. For comparison non-issued VCs or other items are indicated in grey. The bottom panel indicates the positive predictive value of the distance metric at predicting pre-1914 VCs (green) or medals dated 1854 (brown). This equates to the proportion of items with a distance metric of this value or less that match this era or year respectively.

There are two essential questions regarding medals of queried provenance: does the item have a composition as consistent with VCs as other genuine medals, and if so is its composition most similar to those medals dated to the same year. A medal that not only shows a composition similar to other VCs but in particular to those of the same date is likely a stronger candidate for being authentic. Taking the medal found in the river Thames as an example, labelled molVC in Fig. 7, illustrates that its composition not only closely matches that of other medals, but it most closely matches those also dated to 1854. This medal’s composition is therefore consistent with that of an 1854 medal. Only six of the 136 items in the dataset had a metal composition more consistent with 1854 medals. All of these were VCs dated between 1854 and 1879 (two dated 1854, one dated 1855, two dated 1857 and one dated 1879).

**Figure 8:**
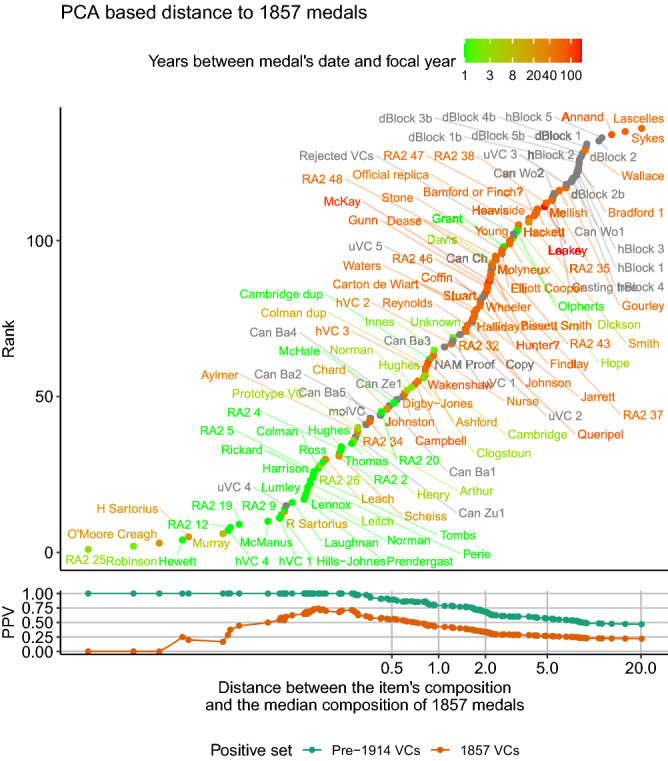
The similarity of items to the group of medals issued in 1857. The weighted squared Euclidian distance between the metal composition of each item and the median composition of all medals issued in 1857 (the medal in question being excluded from the calculation of the median composition if issued in that year). Items in the top panel are shown ranked by their distance and the colour of their points indicates the years between the year in question and the date of the medal. For comparison non-issued VCs or other items are indicated in grey. The bottom panel indicates the positive predictive value of the distance metric at predicting pre-1914 VCs (green) or medals dated 1857 (brown). This equates to the proportion of items with a distance metric of this value or less that match this era or year respectively.

Another medal whose provenance has been queried is that awarded to Lt Harry Prendergast for an action in 1857. With no date visible on its reverse, this medal has a particularly worn and pitted appearance as compared to other medals of the same time-period (Supplementary Fig. S2) leading questions to being raised as to its authenticity in 1983^14^. This medal not only shows a composition consistent with pre-1914 VCs but it is unusually similar to medals dated 1857 (Figs. 5, 8). 16 items showed a composition more similar to the median composition of items dated 1857. However, 9 of these were medals that themselves were dated 1857. Of the remaining items six out of seven were VCs dated between 1858 and 1879. The remaining item showing a more similar composition to the average of 1857 medals was an unissued medal held by Hancocks, the suppliers of the medals, that was in fact inscribed with the date 1857 suggesting it was likely created at this time. Not only does the Prendergast VC therefore show a composition unusually similar to other medals of the same time-period, importantly, nineteen medals dated 1857 showed compositions less similar to the median composition of other 1857 medals than the Prendergast VC. As shown in the hierarchical clustering in Fig. 5 the Prendergast medal has a composition unusually similar to three VCs; two dated 1857 and one dated 1859. Consequently this medal’s composition is consistent with an 1857 medal and it is a better match to medals of this date than 66% of the other medal’s dated to this year.

During the course of this analysis we also noted that the VC awarded to Captain Herbert Clogstoun for his actions during the Indian Mutiny/First War of Independence in 1859 was unusual in its composition for a medal of this time-period (Fig. 5). In the PCA shown in Fig. 6 it shows a composition similar to two replacement VCs (Colman and Cambridge). A subsequent freedom of information request to the National Army Museum (the current holders of the medal) revealed that Hancocks had issued a replacement for this VC in 1938. In 1967, after the Clogstoun family donated the medal to the Museum, Hancocks examined the medal and stated *“that it was not the duplicate cross issued in 1938 but they were unable to state whether or not it was the original cross issued in 1859”*. In terms of Euclidian distance, out of the eight years studied in Fig. 5A, the medal is most similar in composition to those dated 1879 (Table 2, Supplementary Table S2). Though it should be noted this distance is not small at 0.70, as compared to 0.12 for the Thames medal to 1854 medals, and 0.14 for the Prendergast VC to 1857 medals. For context the item with the smallest weighted Euclidian distance that is not a VC item is one of the Dutch shipwreck cannons with a value of 0.26 (Table 2, Supplementary Table S2). The Clogstoun medal clusters in Fig. 5 with two VCs issued in 1941–1942. Consequently further investigation is required, and comparison to other medals dated to around the two dates (1859 and 1938) would likely be particularly informative.

**Table 2:** PCA based distance metrics for selected items to medals of given years.

Item	1854	1855	1857	1858	1879	1916	1917	1918
Clogstoun (1859)	1.11	2.16	0.80	1.12	0.70	2.40	1.40	0.88
Thames VC (1854)	**0.12**	0.91	0.34	0.53	0.27	3.74	2.54	2.00
Chard (1879)	**0.05**	0.62	0.47	0.56	0.44	5.35	3.94	3.17
Dutch cannon	0.76	1.28	0.29	0.41	0.26	2.82	1.70	1.19
Prendergast (1857)	0.69	1.24	**0.14**	0.25	0.18	4.55	3.12	2.51
Copy	2.71	2.02	1.64	1.29	1.86	7.48	5.82	5.39
Unissued VC (1857)	0.73	1.33	**0.10**	0.25	0.15	4.71	3.26	2.55

Only results for years with at least four medals are shown. The cannon metrics shown are for the Dutch shipwreck cannon with the smallest distance metric in one of the years. Distances less than 0.15 are highlighted.

As discussed above, a further medal that has previously had its authenticity queried was that awarded to Lieutenant John Chard during his actions at Rorke’s Drift in 1879. Although previously thought to be a replica, prior metallurgical analysis in the 90 s suggested it was potentially the original medal. The analysis here confirms that its composition is similar to other medals issued prior to 1914 (Table 2, Supplementary Table S2). Of the years examined it in fact shows an unusually close match to medals issued in 1854 (weighted Euclidian distance of 0.049) with comparatively less similarity to those issued in 1879 (0.44).

Of note the unissued VC held by Hancock’s inscribed with the year 1857 had a composition most similar to other medals also dated 1857 (weighted Euclidian distance of 0.10, Table 2), and the VC copy analysed by the Royal Armouries, that contained an unusually high proportion of tin (Supplementary Fig. S3), had a composition that was not similar to any set of medals (Table 2, smallest weighted Euclidian distance of 1.29). Composition analysis is therefore likely an effective approach to inform the potential authenticity of VCs.

## Discussion

A previous study suggested that the source material for Victoria Crosses changed following the increased demands for medals at the beginning of the First World War^7^, and our analysis is consistent with this. A clear split in the composition of medals issued before and after 1914 is observed. However, we have demonstrated that, even within these groups, medals can be further broken down into sub-groups of medals showing differences in their composition to other medals of the same era. It is possible this is a result of different sources but given the relative consistency of medals within eras may also simply reflect the fact that VCs are created in batches. For example, both simple pouring and centrifugal casting have been used in creation of the medals, and different casting approaches at different times may lead to inconsistencies between batches even if the source material is the same. Consistent with this, these sub-groups generally show similarities in the dates of the respective VCs, but it should be noted that medals that are cast together may not always be issued together. As a result medals issued for actions in different years may potentially have still been cast together and therefore show similar compositions. An example of this may be the Chard VC whose composition most closely matches earlier medals.

Another potential driver of differences in the composition between groups of medals is quantification error. Inconsistencies in the states of the medals, the shape of the measured area or variation in the composition across a medal could all cause artefactual differences. Reassuringly, however, greater variation was observed between the measurements of different medals than repeated measurements of the same medal between or within studies. Furthermore, the medal that had potentially been in the river Thames for over a century was still a very close match to medals dated to the same year. Consequently, there was not strong evidence that quantification error was the main driver of the differences observed between the medals.

Cannon held at Woolwich, often referred to as the “VC guns”, are widely thought to be the source of many issued VCs, though which and how many is not known. Comparison of the composition of these guns to this large set of medals highlights that they are in fact not a close match to any of the VCs nor the sets of metal blocks held by the UK Ministry of Defence at Donnington and by Hancocks. The cannon from Dutch eighteenth century shipwrecks were in fact closer matches, at least to pre-1914 VCs. So although the metal stored at Donnington is likely to be the source of the metal at Hancocks, this analysis raises the question as to whether these cannon are indeed the source material of these blocks or indeed any issued VCs. One caveat to this is the composition of a cannon likely differs along its length. Although the Donnington and Hancocks blocks generally show high consistency in their compositions it is possible that the remaining sections of cannon do not match the composition of the sections that have been removed.

Using these data we investigated three VCs whose authenticity have been at one stage queried. As previously suggested by the initial analysis by the Royal Armouries with their smaller set of VCs, the medal ascribed to Lt John Chard is a very good match to other early VCs. Consequently these data support the idea that this medal is in fact original. Despite its worn and pitted appearance the Prendergast VC is also a very close match in terms of composition to other early VCs, with its closest matches being two other medals also dated to 1857, one that has been held through this time by Hancocks themselves. Importantly both the Chard and Prendergast medals were first suggested to not be authentic prior to the earliest XRF analyses of VCs, so that the composition of VCs was unknown at the time. This means not only was the authenticity queried on appearance alone, it would have made making copies with such close matches to the composition of other VCs of the same time-period not only extremely difficult, but likely unwarranted given composition analysis had yet to begun to be used to authenticate VCs. It therefore seems unlikely any copies would be such good matches to VCs of the same period. Although the Chard medal has changed ownership, the Prendergast medal has been held directly by the Prendergast family or on long-term loan to the National Army and Royal Engineers Museums since being awarded.

The medal found in the River Thames in 2015 is slightly different in that in theory the composition of VCs was known at the time of its discovery. However, its composition is an unusually close match to other medals of the same time period suggesting it is also likely an authentic VC. During the course of this analysis one named medal was identified whose composition was unusual for VCs of its time-period; the medal awarded to Captain Herbert Clogstoun for his actions during the Indian Mutiny/First War of Independence in 1859. This medal is a very poor match in terms of composition to all other VCs of this period. Subsequent investigation highlighted that an official copy of this medal had been produced, though after its examination Hancocks have stated the medal held at the National Army Museum is not this later copy. Although they stated it was unclear if it was the original medal, this current analysis suggests this may not be the case. Further comparison to other medals issued in the two possible time periods would be informative.

The method presented here for determining how similar a medal might be to others of a given year could likely be improved further, in particular if a larger set of composition data can be compiled. For example, currently all medals of a given year are treated as one group, despite our analysis demonstrating that even within years different groups of medals can be observed. Consequently calculating distances to these sub-groups of medals of the same or similar date separately, may improve the performance of this analysis. Though this would depend on having a sufficient number of medals to accurately define these sub-clusters and their average composition.

We have therefore analysed XRF data for the largest collection of VC medals to date. These data provide further understandings of the history and source of this medal and the authenticity of VCs.

## Methods

The Royal Armouries XRF data were collected under laboratory conditions using a Kevex 7500 XRF spectrometer, the methods being supervised by the Royal Armories and Tower of London authorities. For medals, measurements were taken on the inscribed back. The more recent data (2016–2018) were all collected using the PXRF Bruker Tracer III^9^ using its yellow filter (Ti and Al) with the voltage at 40 kV and the current at 10 μA. Empirical calibrations for copper, CU1 CFZ, were used as available on the software. Medals were placed on the sample table of the spectrometer and exposed to X-ray beams for 120 s. Each medal was sampled front and rear and as close to the centre as possible. Cannons and the Donnington cascabel piece were examined using the PXRF in the handheld mode and at various points on their surfaces; again, using exposures of 120 s. After the data were collected, they were refined using empirical calibrations for copper alloys. As well as removing the eight VCs from this dataset with a low correlation between their readings as described (Pearson’s r < 0.95 between (natural) log transformed composition values), one of the VC gun measurements in this dataset was also excluded from all analyses due to an unusually high iron reading compared to the other measurements of the same item.

The Royal Armouries unpublished reports contained XRF data for a further 68 VCs, not including the 8 VCs measured by both studies. Two of these additional VCs could not be included (RM 1854 and HVC 1854) as their zinc compositions were not recorded by the Royal Armouries. This was attributed to the presence of platinum in the samples. Many of the VCs in the RA analysis were only annotated with the year and museum at which it was held. For most this was sufficient to determine to whom it had been awarded but for the remainder they are simply labelled in this analysis as RA followed by a unique number. One medal from 1914 labelled in the analysis as being awarded to “Cpt WH Johnson” was determined to be a misspelling and relabelled Johnston. Further to these VCs the RA report contained XRF results for an official replica VC dated 1901, five unissued VCs held by Hancocks (one dated 1857), a block of metal made by melting down various VCs rejected after casting, a casting tree remaining after casting a group of medals, five blocks of metal supplied by the Ministry of Defence to Hancocks for creating the VCs and two blocks of metal held at the MoD base in Donnington thought to be the source of the VC metal. Finally the report contained results for two cannon held at Woolwich which were stated in the report as “reputed to be those from whose metal the first VCs were cast”.

The final source of XRF data in this analysis was a previous study of cannon found on Dutch shipwrecks off of the coast of Australia^12^. These XRF results were used as controls when examining the metal content of the potential source cannon. The final list of 136 items analysed in this study can be found in Supplementary Table S1.

All statistical analysis was carried out in R. Heatmaps were plotted using ComplexHeatmap^15^. In all analyses the composition metrics were restricted to the six most commonly occurring metals (iron, copper, zinc, lead, tin and arsenic). The scale function in R was used to transform the composition metrics across items by metal prior to plotting the heatmaps in Fig. 5 and Supplementary Fig. S3 to enable the direct comparison of the relative composition of metals.

The Gaussian finite mixture modelling was performed using the mclust package^16^ with the number of mixing components and the covariance parameterization selected using the Bayesian Information Criterion (BIC) approach implemented within the software. The VVE mclust model with a four-component mixture had the highest BIC and was used in downstream analyses. The contour plot of estimated mixture densities and uncertainty boundaries were generated using the MclustDR function and the mclustBootstrapLRT function was used to carry out the likelihood ratio tests of the number of components.

The principal component analysis was carried out using the prcomp function in R with the centre and scale parameters set to true. The weighted squared Euclidian distance of items (i) to the median PC coordinates of medals from a given year was calculated as:

$${weighted squared Euclidian distance}_{ij}=\left({\left({PC1}_{i}-{meanPC1}_{j}\right)}^{2}\times importPC1\right)+\cdots + \left({\left({PC6}_{i}-{meanPC6}_{j}\right)}^{2}\times importPC6\right)$$

where PC1_i_ is the coordinate of item I on PC1, meanPC1_j_ is the mean coordinate on PC1 of all VC medals from year j, and importPC1 is the proportion of variance explained by the first PC. This method has the advantage over calculating the squared Euclidan distance directly from the raw metal measurements in that metals that better distinguish between the items are effectively upweighted in the final metric, whereas the PCs that explain little variance contribute little to the differences in the final distance metric. Distance metrics for years with at least four medals are shown in Figs. 7, 8 and Supplementary Figs. S4 to S9.

For comparison we also calculated this metric using the Gaussian mixture modelling results using the coordinates on the three reduced dimensions obtained when run across all the items and the corresponding eigenvalues used as the weights. The results were broadly comparable to the PCA based distances (Supplementary Table S2) and did not appear to improve the positive predictive values observed (Supplementary Figs. S10 to S17).

For the purpose of calculating PPV, i.e. the proportion of items below each distance cutoff that were VCs of a given era or year, the Thames, prototype and unissued VC dated 1857 were treated as true positives in both the era and year calculations. It should be noted that the PPV will often be an underestimate as many items, such as the remaining unissued VCs, are treated as false positives due to a lack of dates associated with their manufacture.

Precise values for the composition of medals have been deliberately left out of figures due to a concern that making this data available could reduce the future use of XRF data in assessing the authenticity of medals.

## Supplementary information

Supplementary Information 1.

Supplementary Information 2.

Supplementary Information 3.
