# Supplementary material for: Investigating the origin and authenticity of Victoria Cross medals using X-ray fluorescence spectrometry
Source: Sci Rep. 2020 Nov 19;10:19953. doi: 10.1038/s41598-020-76783-y (PMC7678865; doi:10.1038/s41598-020-76783-y)
Supplement: Supplementary file 3 — Supplementary Information 3. [file 41598_2020_76783_MOESM3_ESM.docx]

SUPPLEMENTARY INFORMATION

Investigating the origin and authenticity of Victoria Cross Medals using X-ray fluorescence spectrometry

Andrew Marriott^1*^, James G. D. Prendergast^2*^

^1^School of History, Classics and Archaeology, Armstrong Building, Newcastle University, NE7 1RU, United Kingdom

^2^The Roslin Institute, University of Edinburgh, Easter Bush Campus, Midlothian, EH25 9RG, United Kingdom

* Correspondence to:

AM – agmarriott@aol.com

JGDP – james.prendergast@roslin.ed.ac.uk


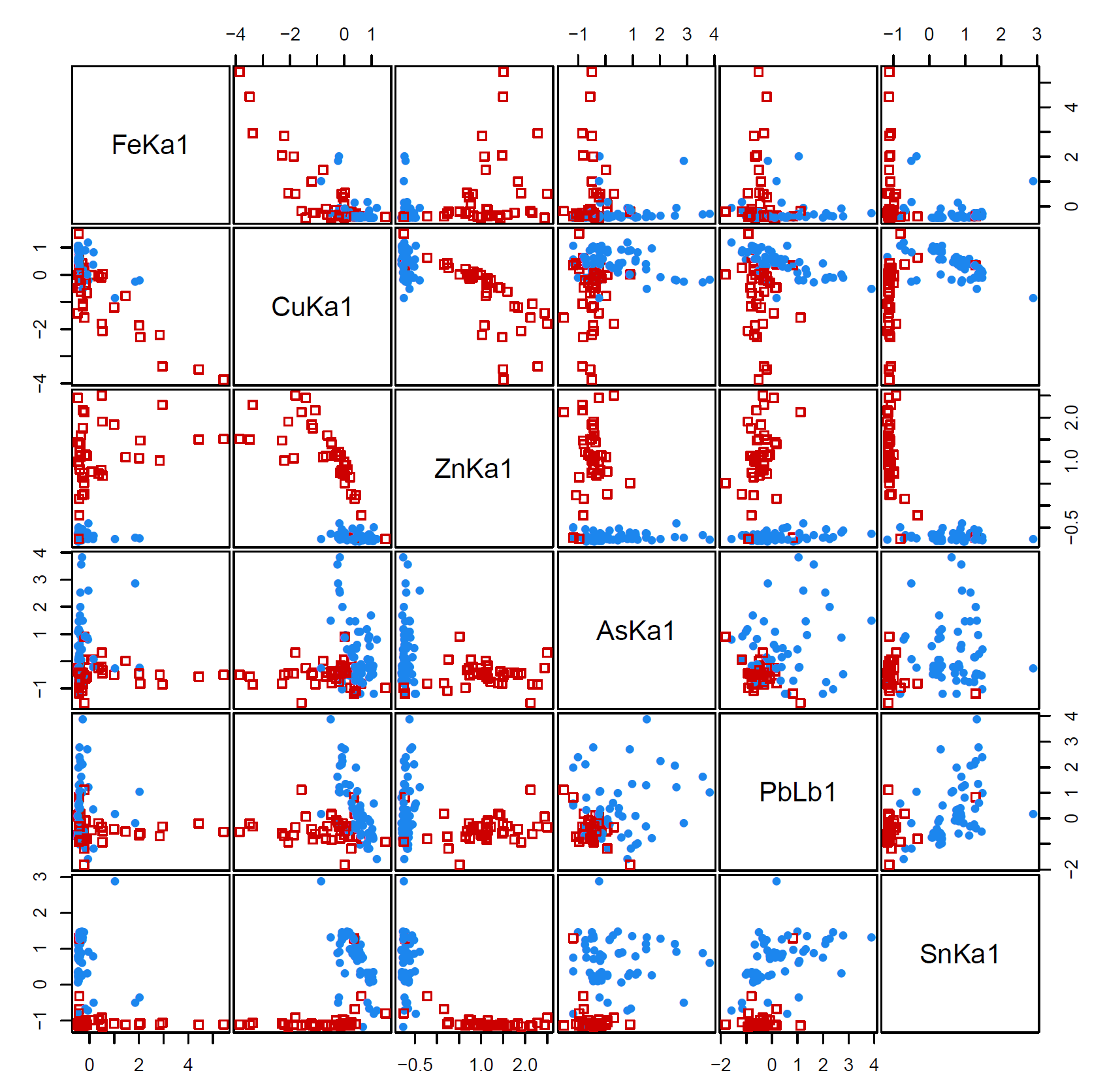


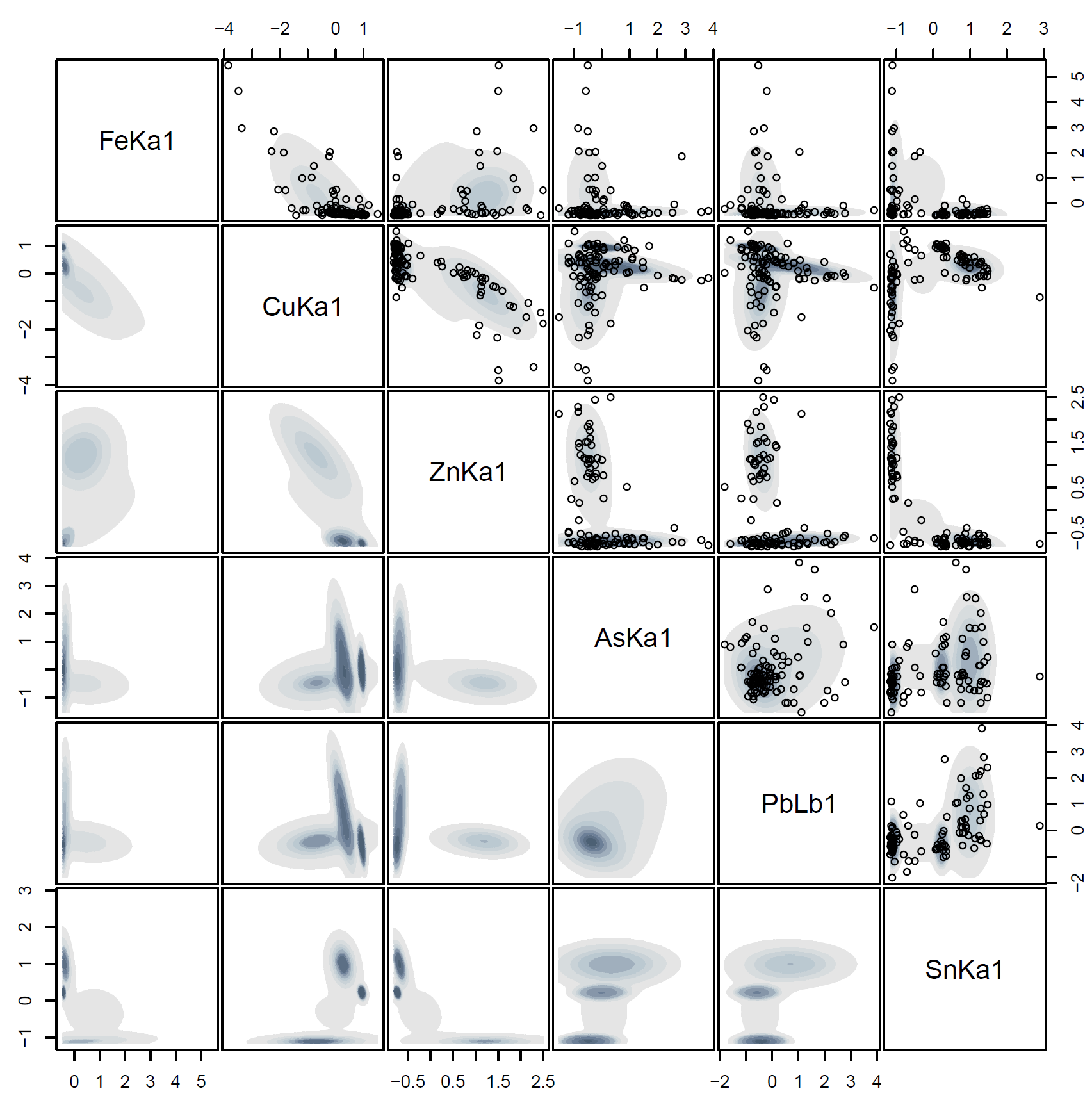


**Supplementary Figure S1.** The metal composition of VC medals. A) Standardised metal compositions. Blue circles – pre-1914 VCs, red open squares – post-1914 VCs B) The Gaussian finite mixture modelling density estimates


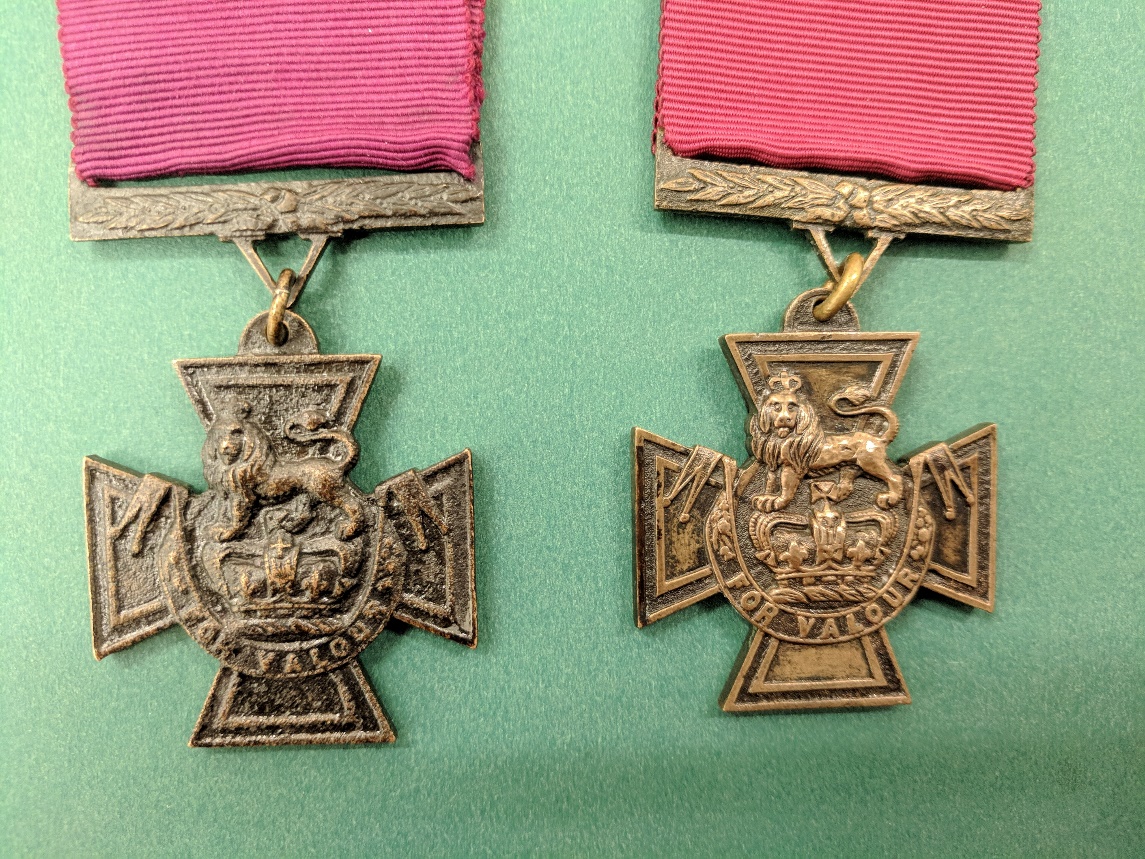

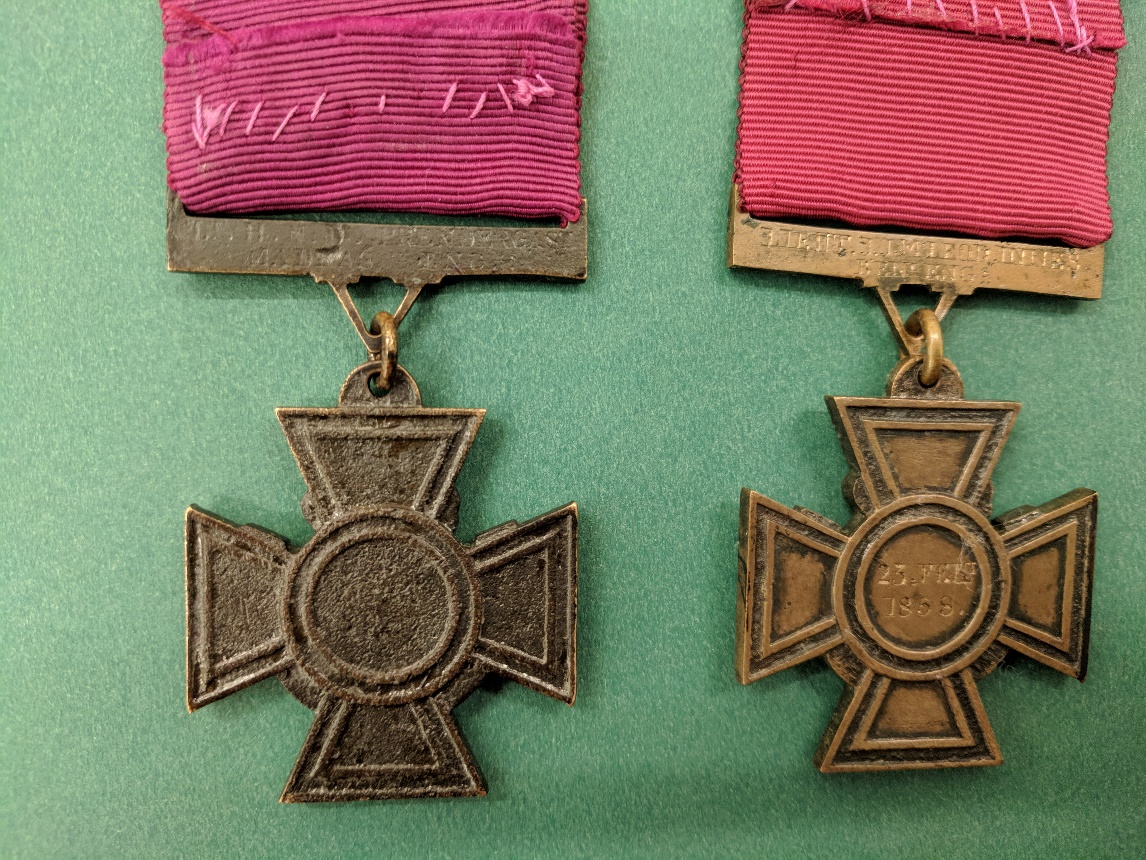


**Supplementary Figure S2.** The obverse (top) and reverse of the Prendergast (left) and McLeod Innes VCs that were both awarded for actions during the Indian Mutiny.


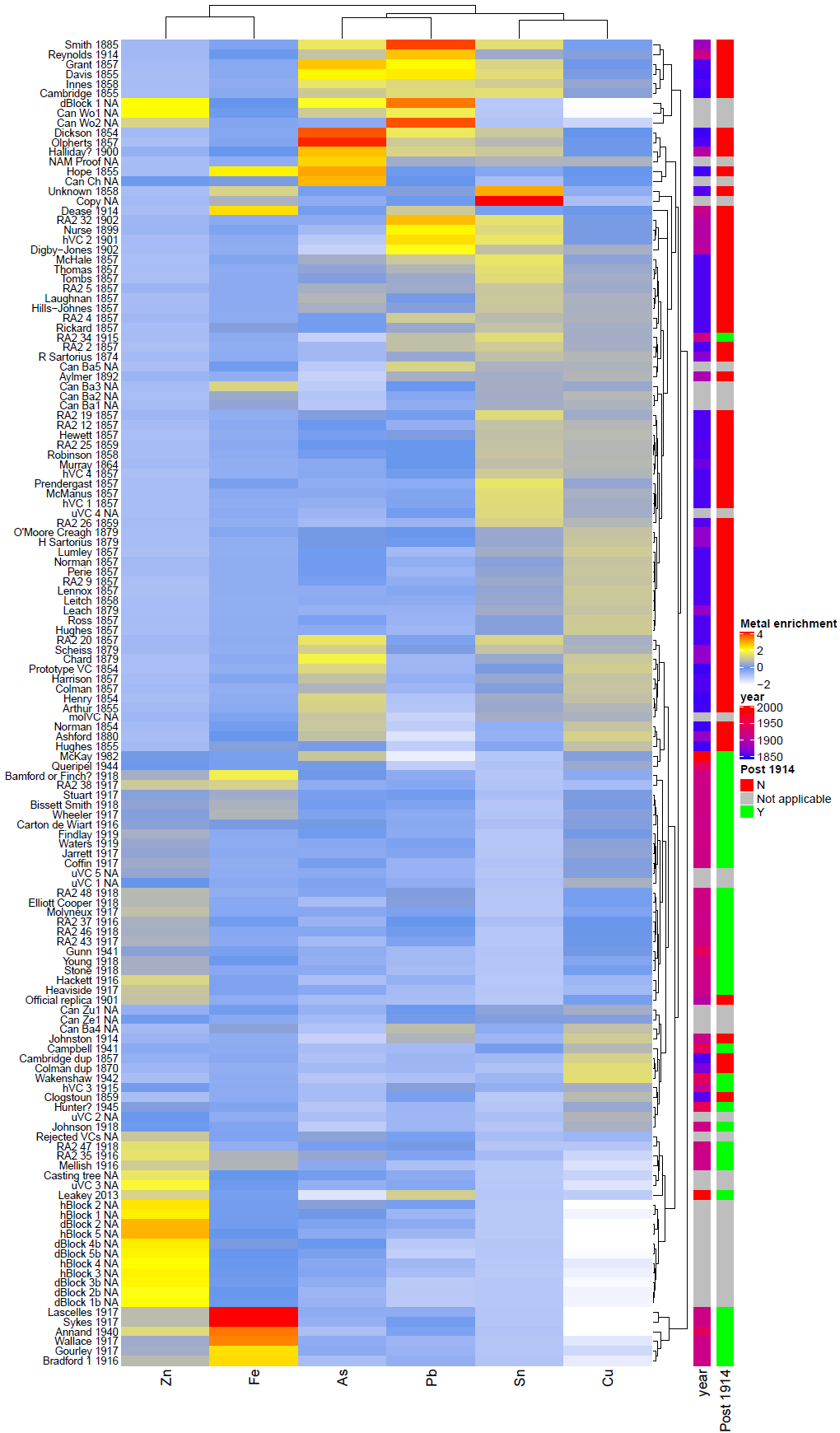


**Supplementary Figure S3.** Hierarchical clustering of all items in the study according to their composition.


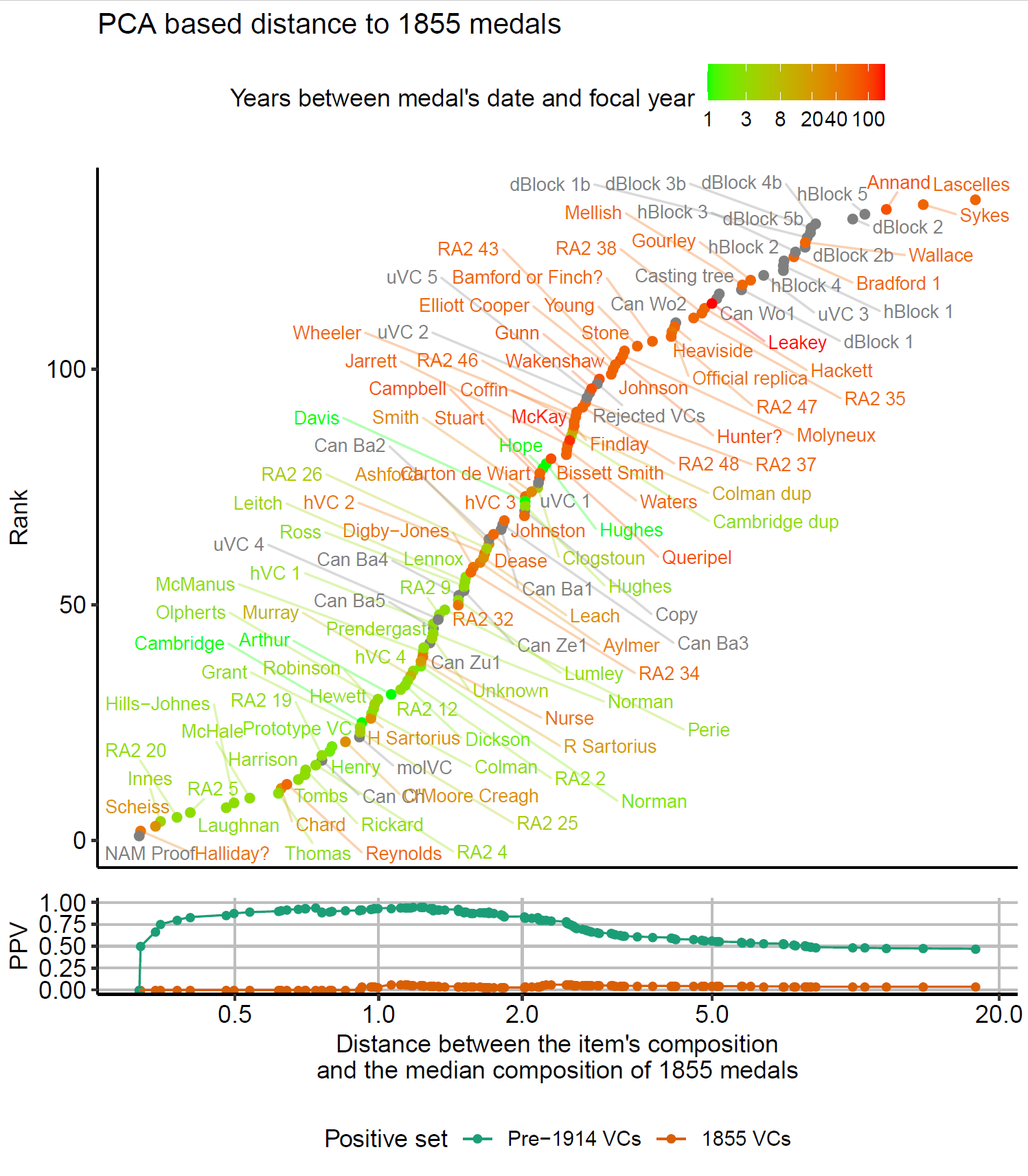


**Supplementary Figure S4*.*** The PCA based distance metrics to medals dated 1855.


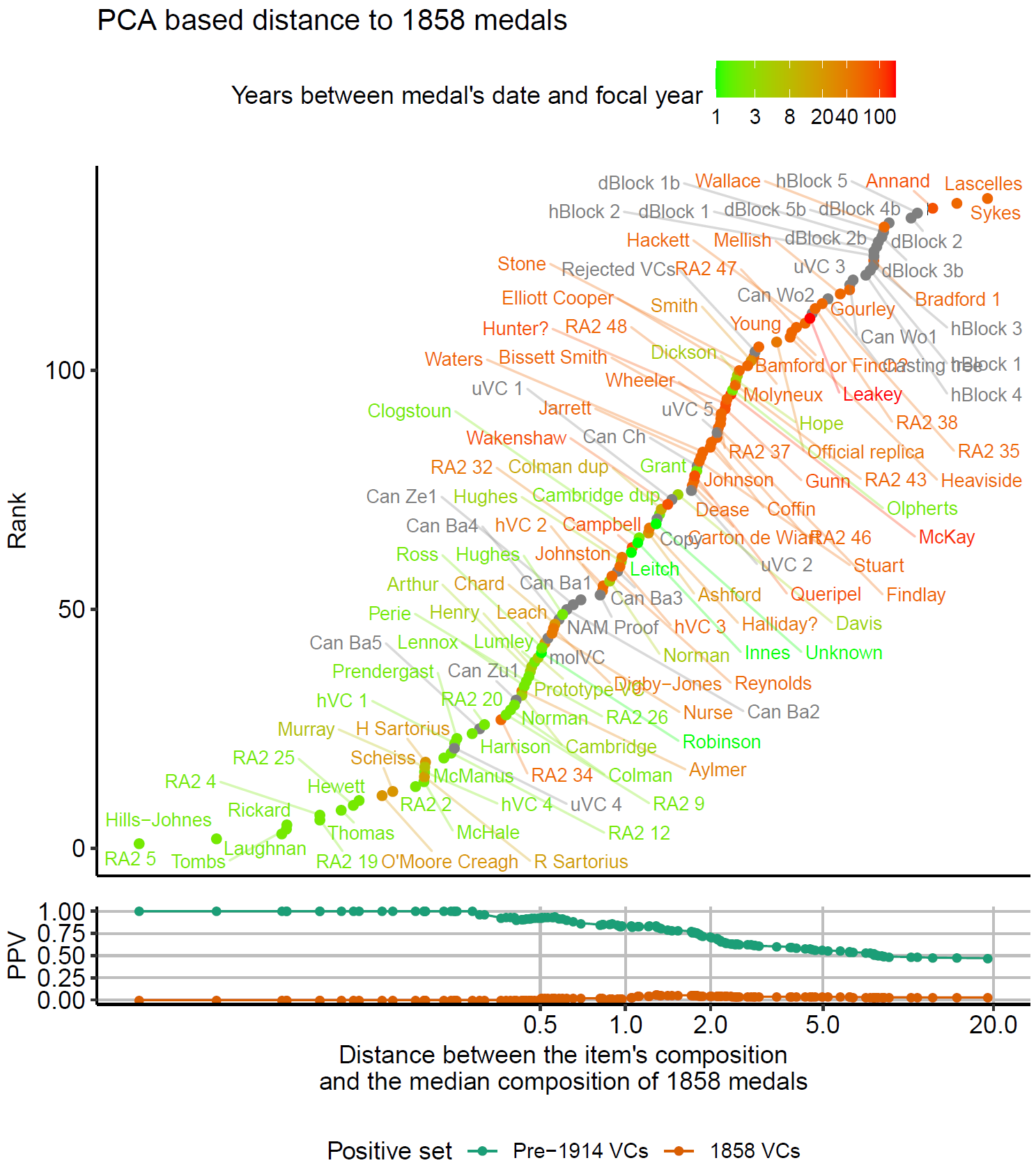


**Supplementary Figure S5*.*** The PCA based distance metrics to medals dated 1858.


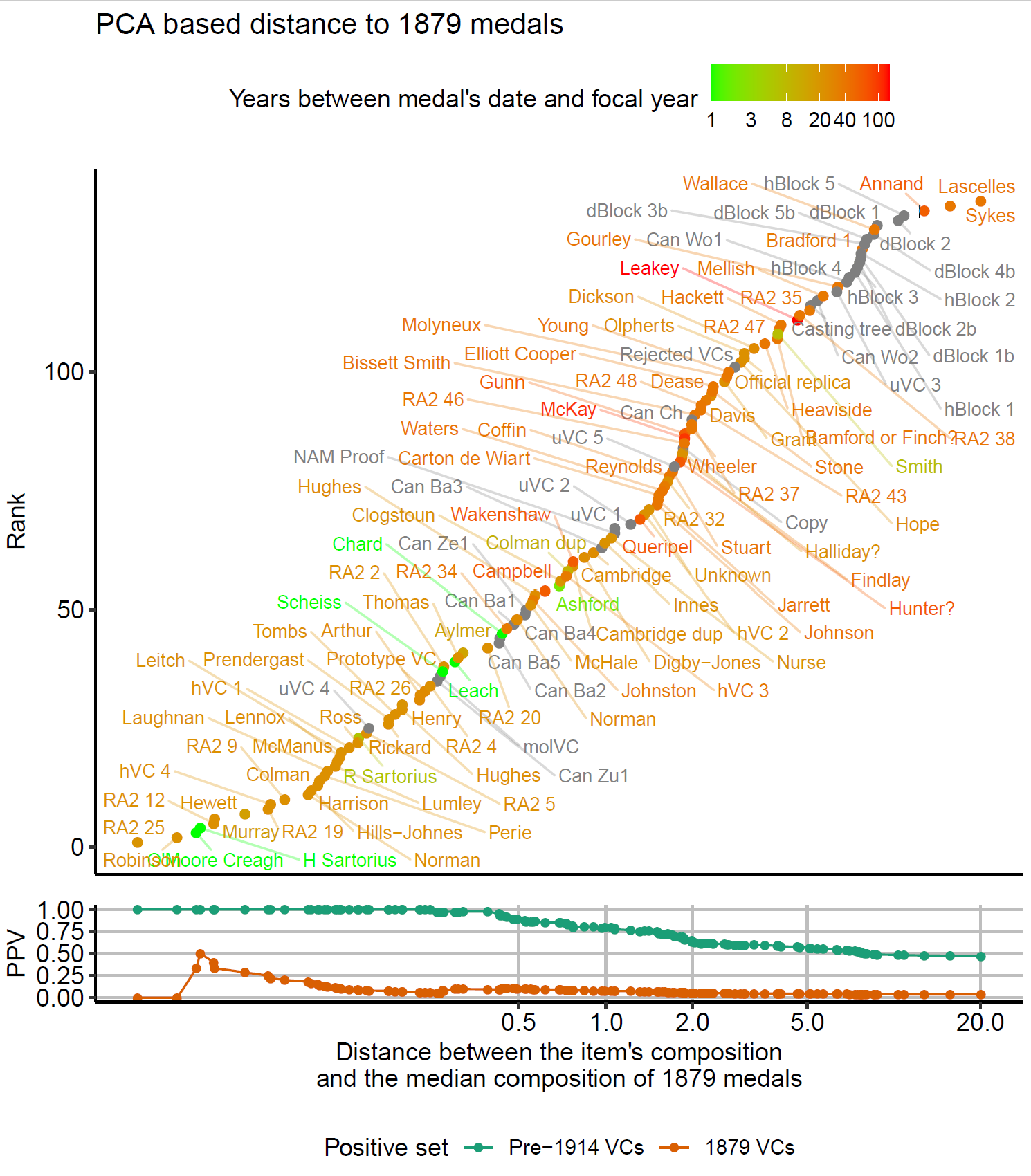


**Supplementary Figure S6*.*** The PCA based distance metrics to medals dated 1879.


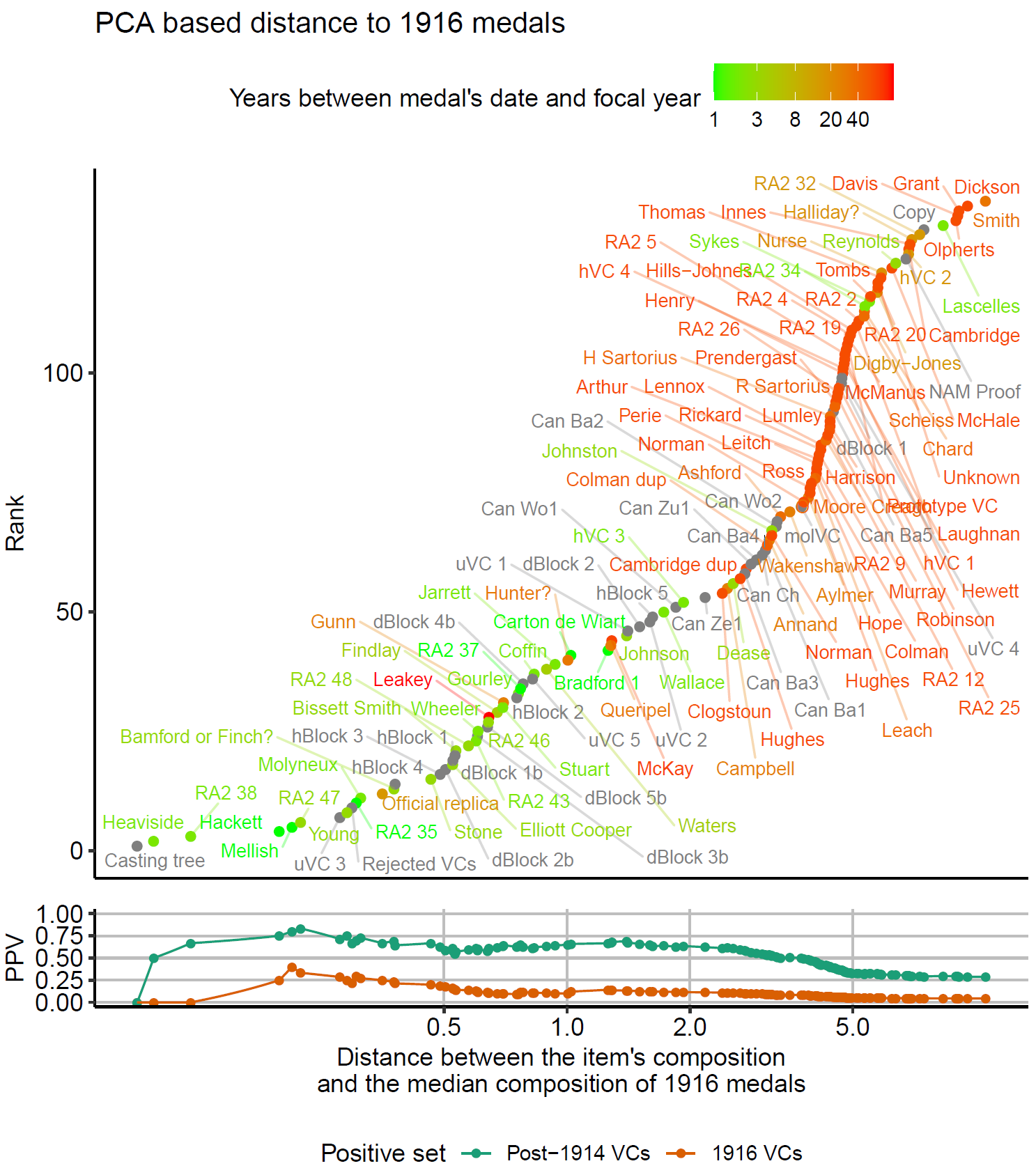


**Supplementary Figure S7*.*** The PCA based distance metrics to medals dated 1916.


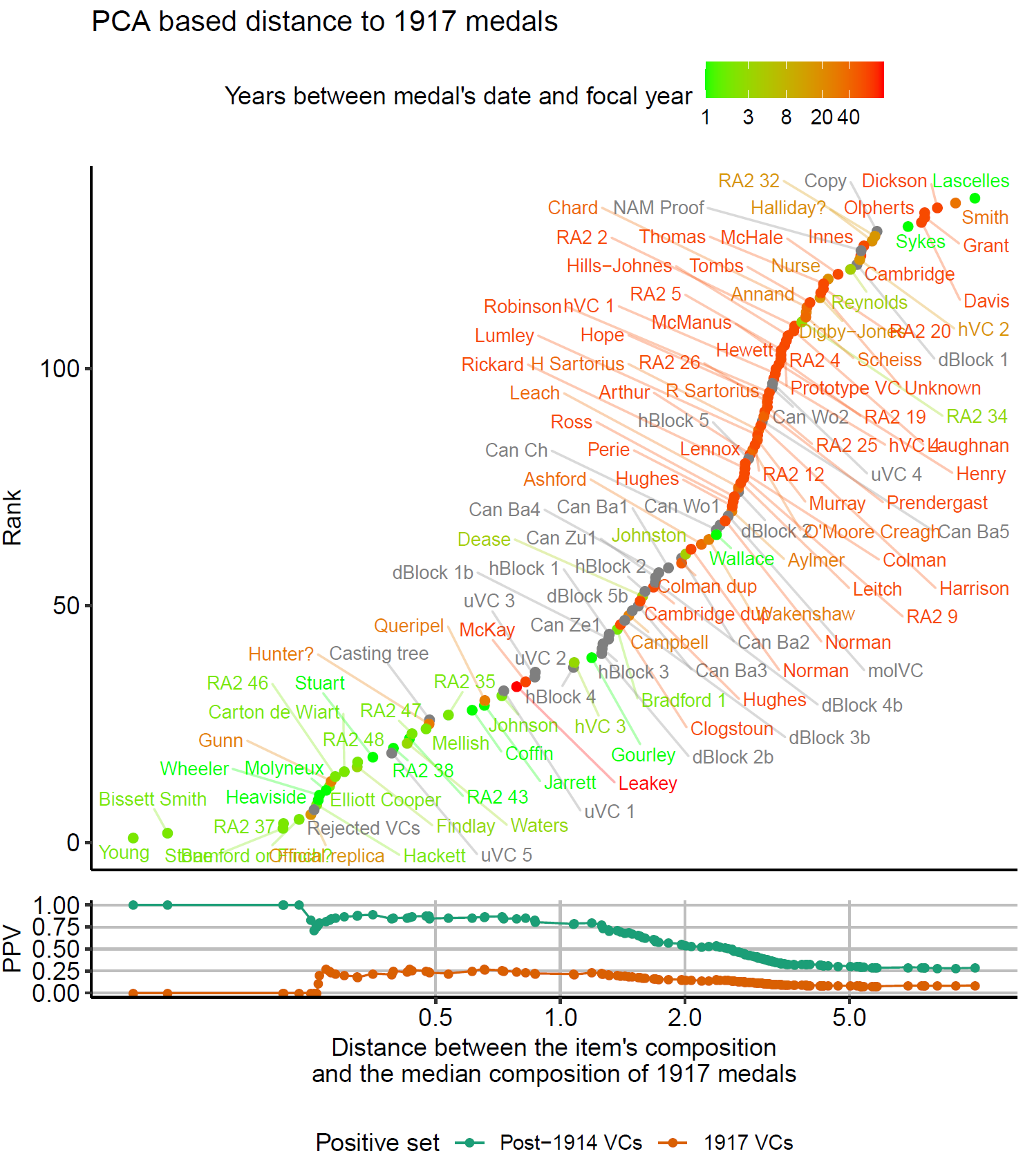


**Supplementary Figure S8*.*** The PCA based distance metrics to medals dated 1917.


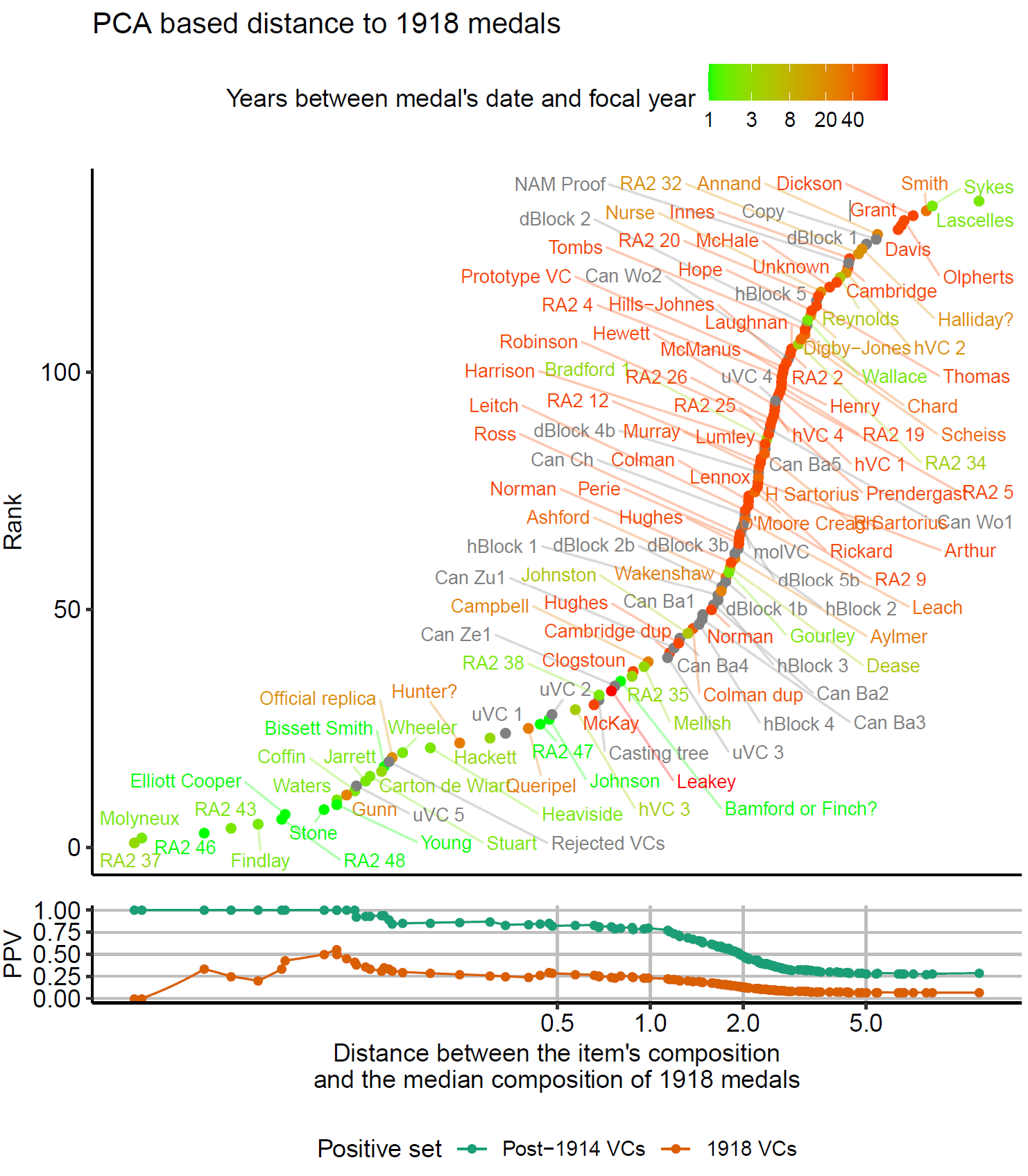


**Supplementary Figure S9*.*** The PCA based distance metrics to medals dated 1918.


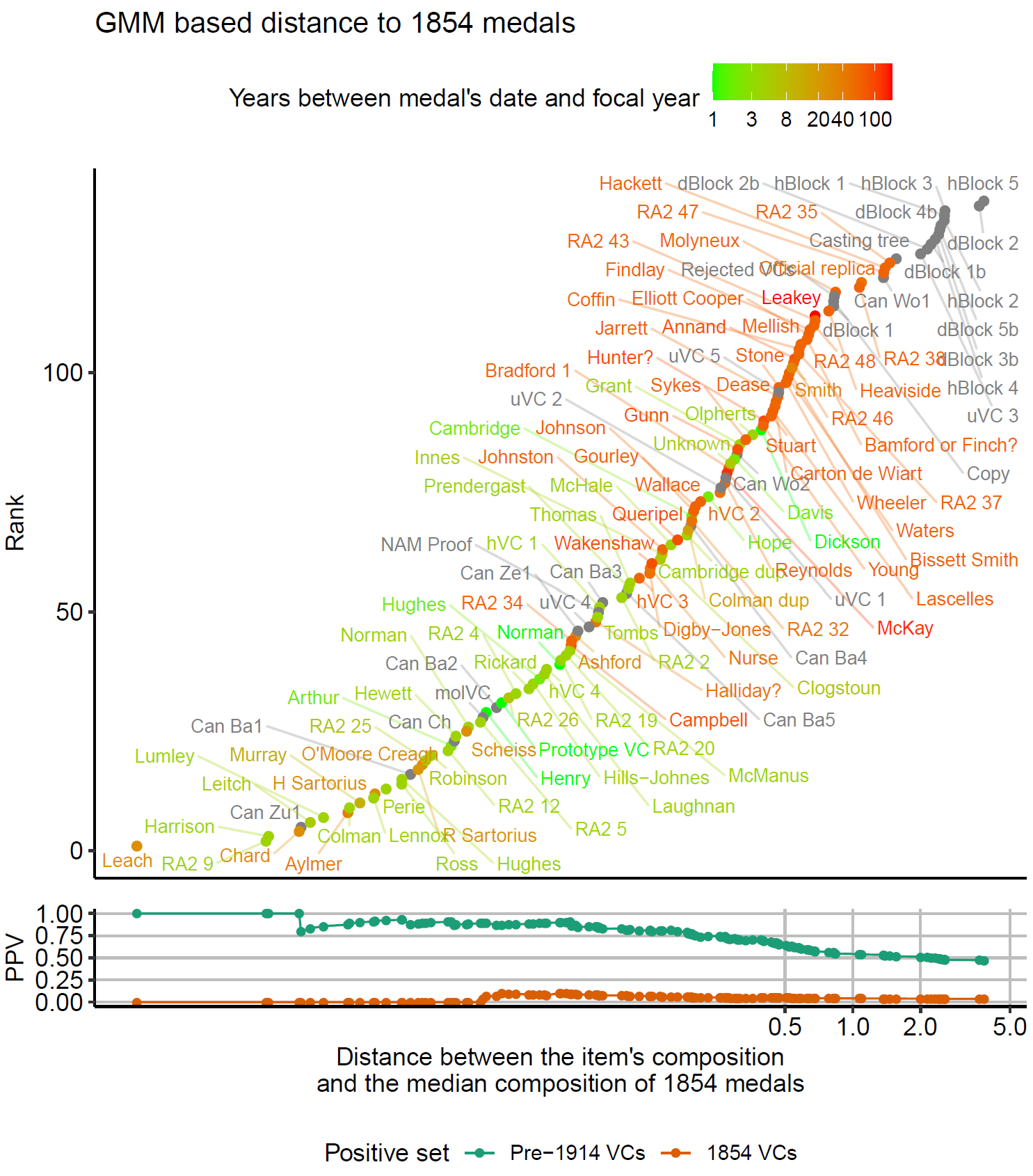


**Supplementary Figure S10*.*** The GMM based distance metrics to medals dated 1854.


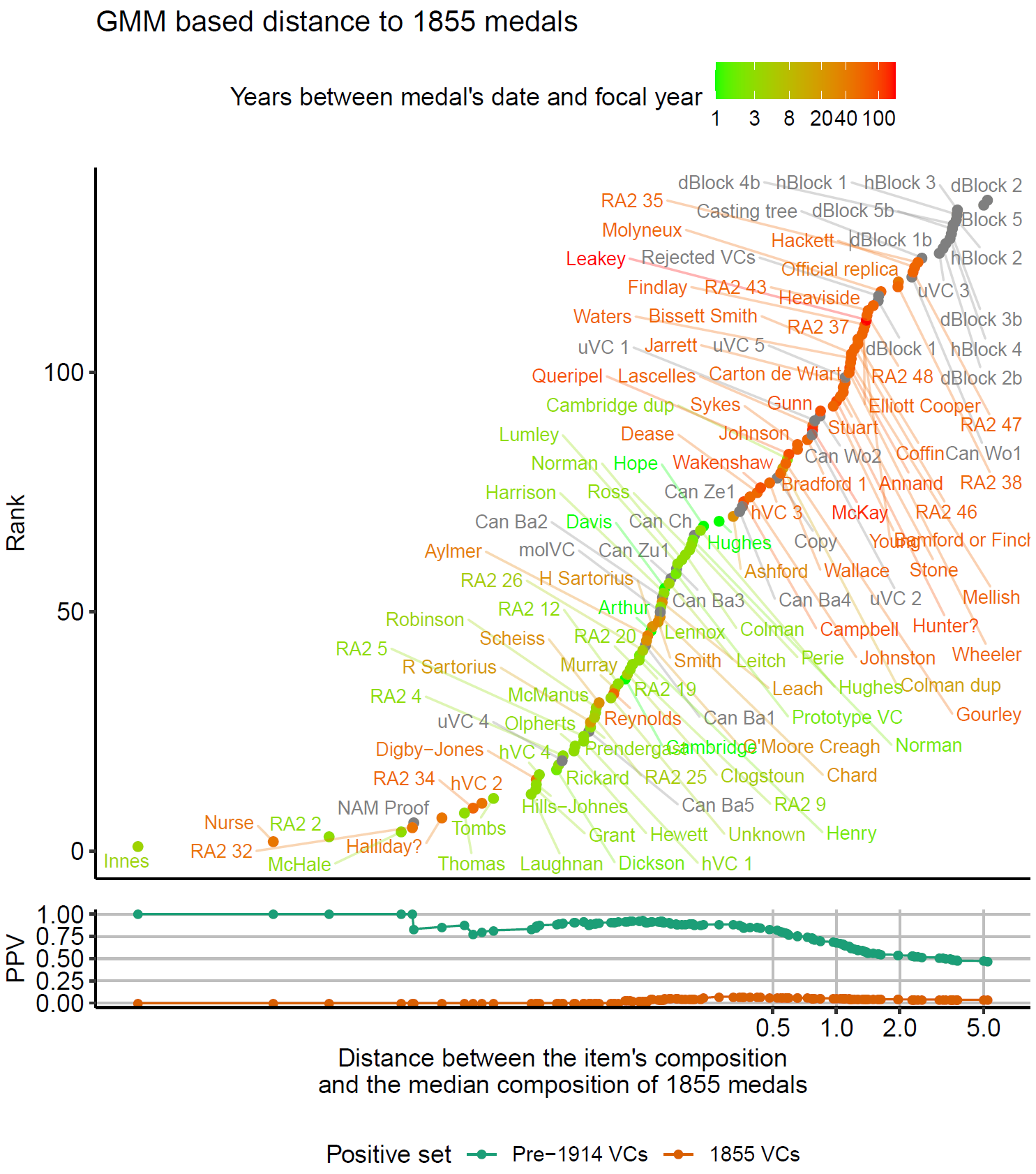


**Supplementary Figure S11*.*** The GMM based distance metrics to medals dated 1855.


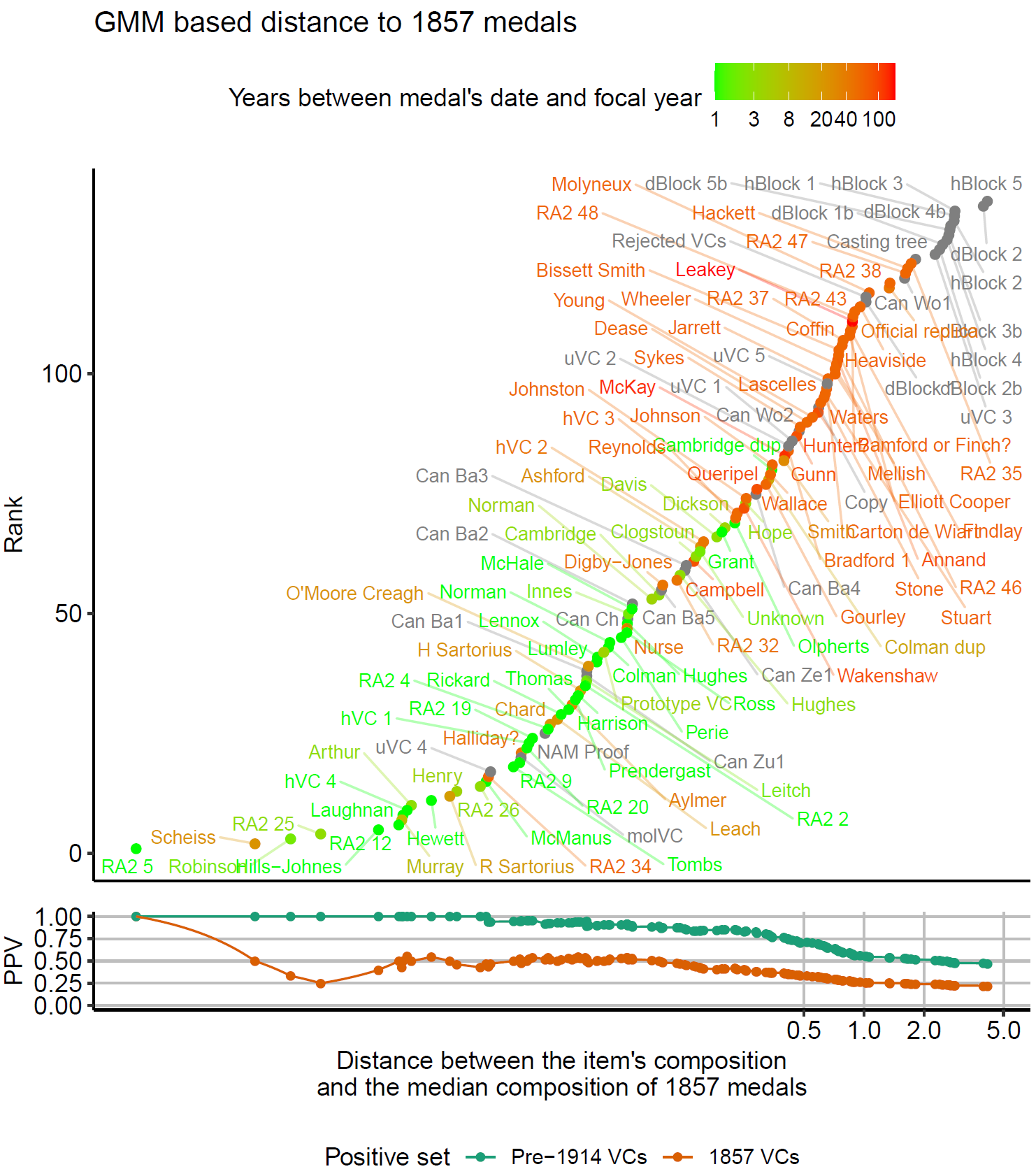


**Supplementary Figure S12*.*** The GMM based distance metrics to medals dated 1857.


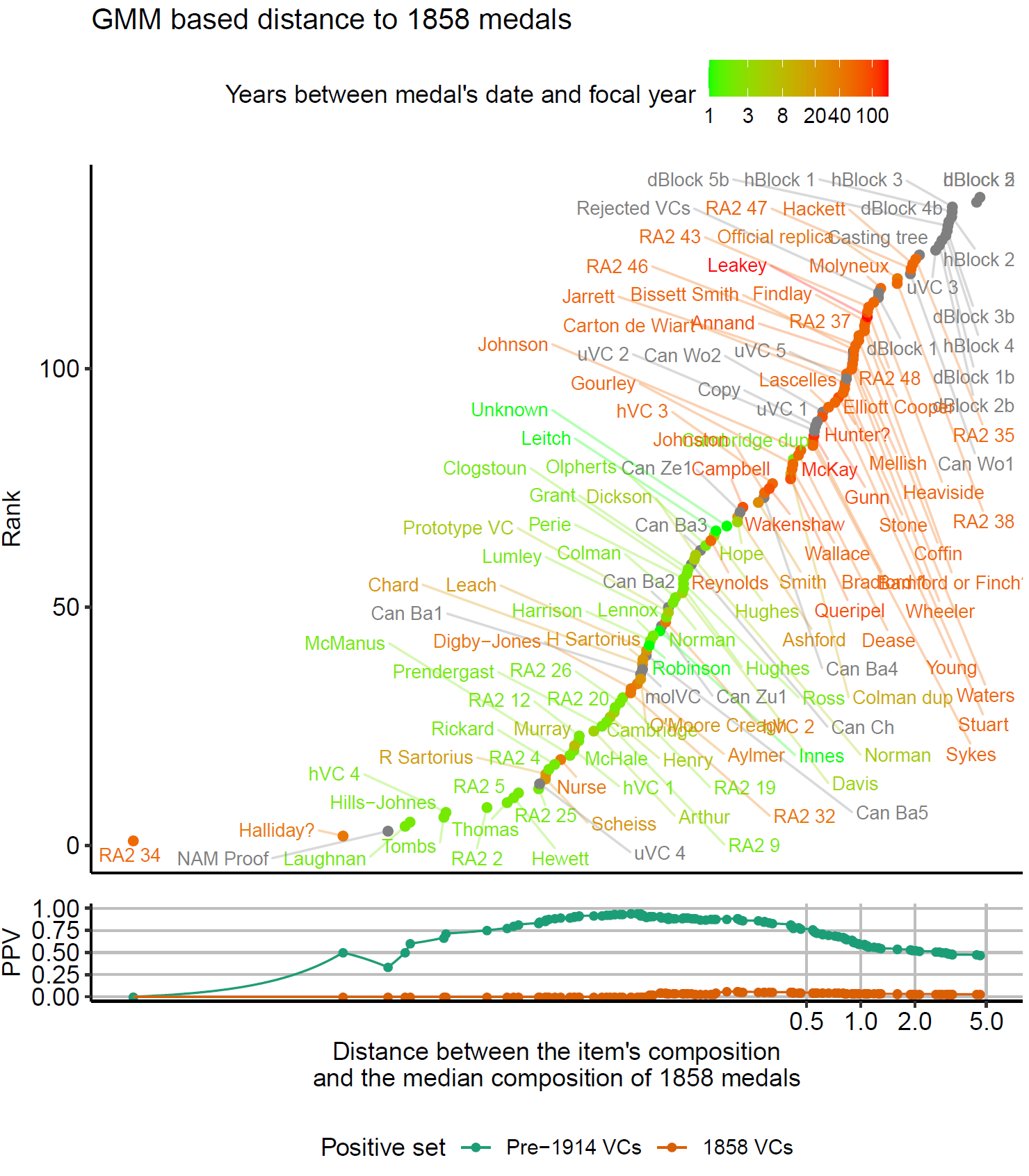


**Supplementary Figure S13*.*** The GMM based distance metrics to medals dated 1858.


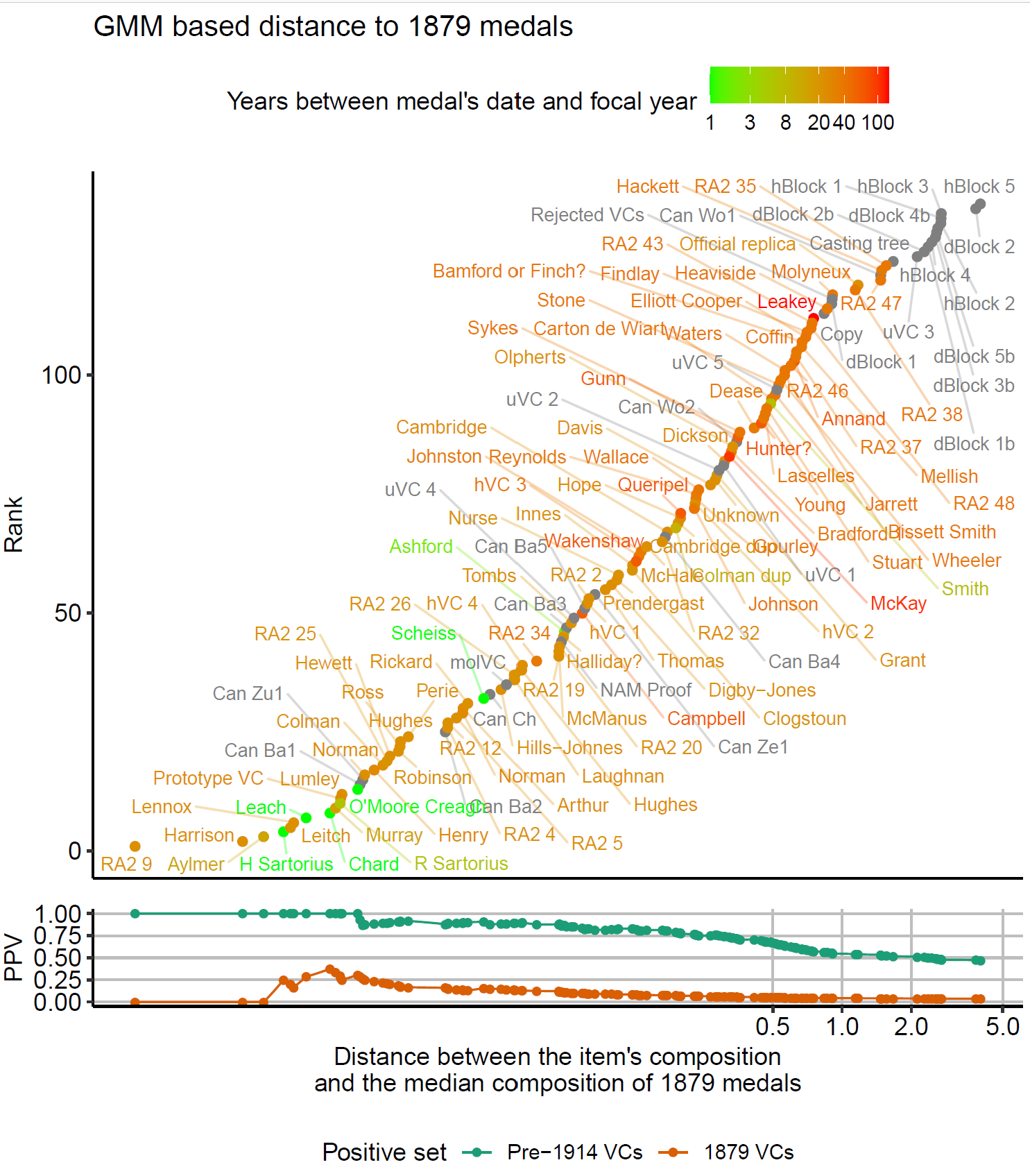


**Supplementary Figure S14*.*** The GMM based distance metrics to medals dated 1879.


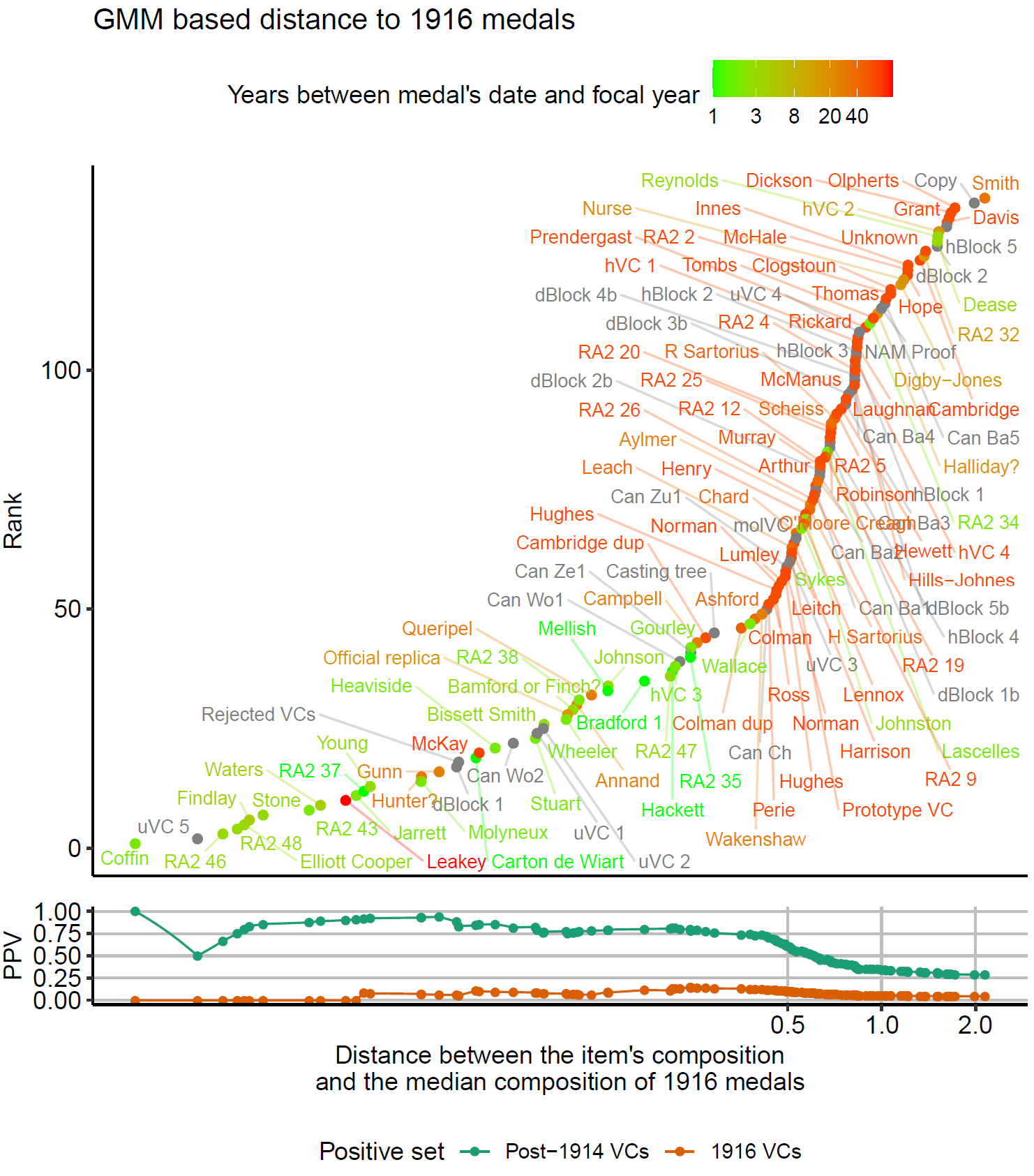


**Supplementary Figure S15*.*** The GMM based distance metrics to medals dated 1916.


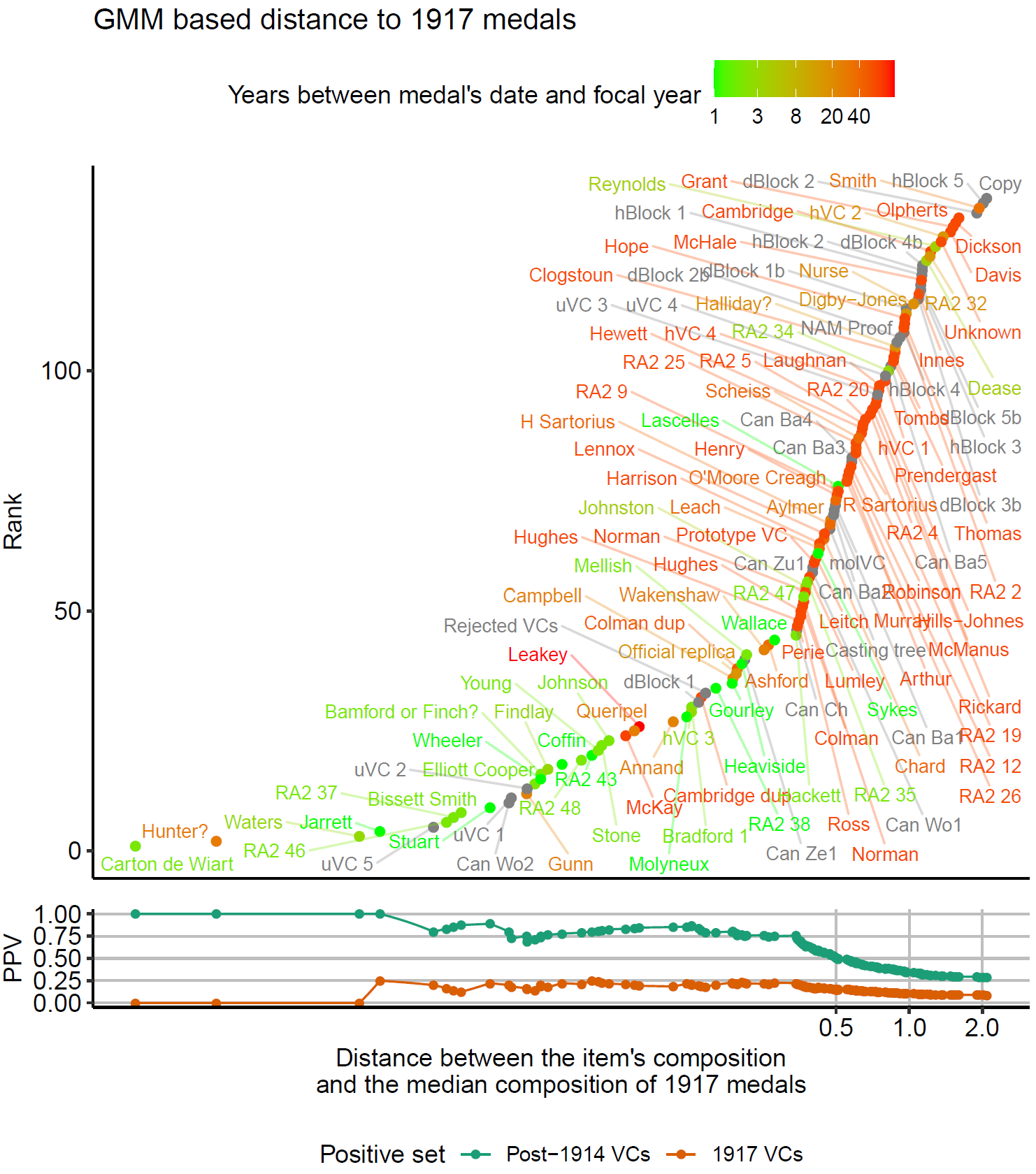


**Supplementary Figure S16*.*** The GMM based distance metrics to medals dated 1917.


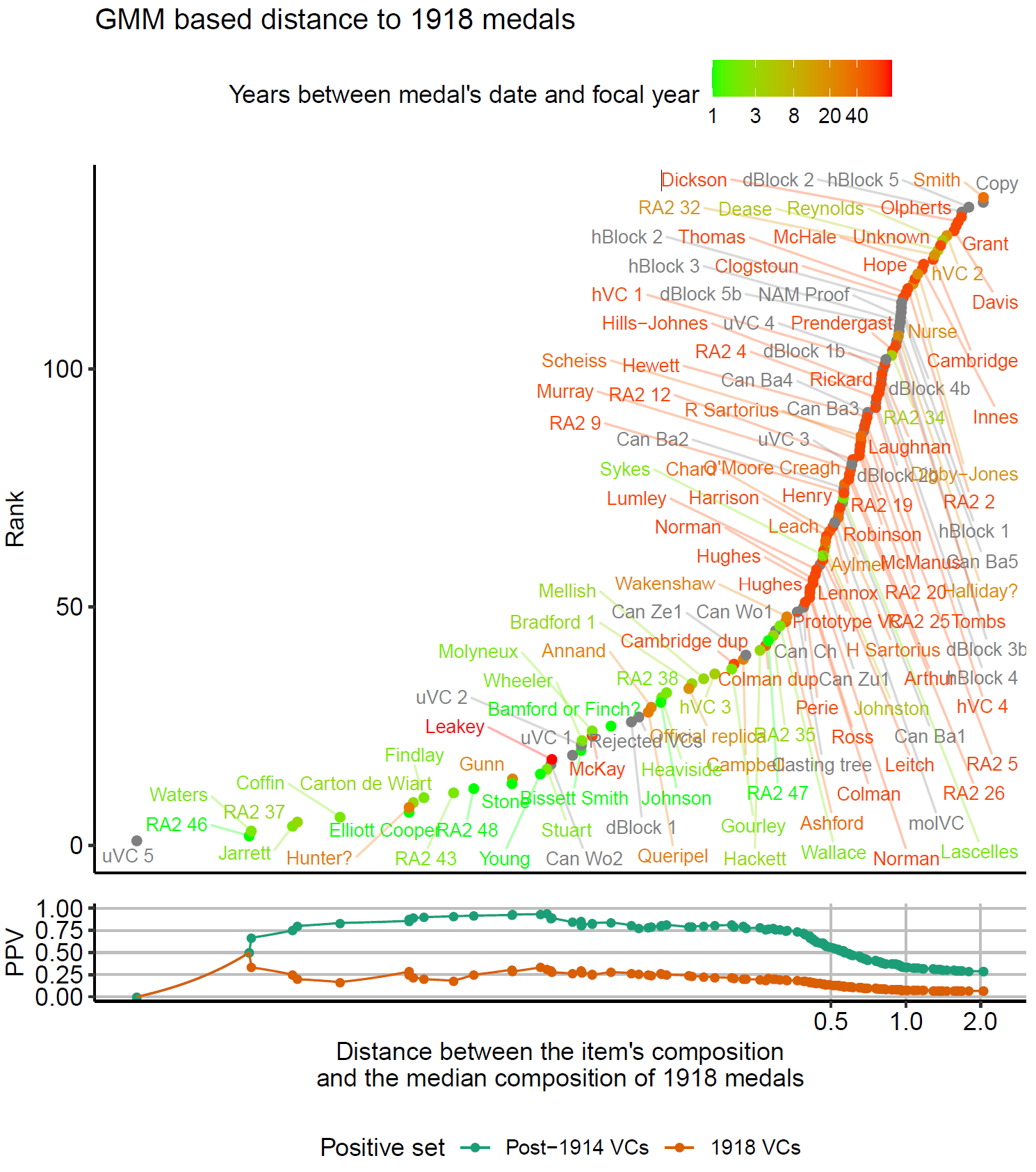


**Supplementary Figure S17*.*** The GMM based distance metrics to medals dated 1918.
